# Post-translational dysregulation of glucose uptake during exhaustive cycling exercise in vastus lateralis muscle of healthy homozygous carriers of the ACE deletion allele

**DOI:** 10.3389/fphys.2022.933792

**Published:** 2022-09-06

**Authors:** Martin Flück, David Vaughan, Jörn Rittweger, Marie-Noëlle Giraud

**Affiliations:** ^1^ Institute for Biomedical Research Into Human Movement and Health, Manchester Metropolitan University, Manchester, United Kingdom; ^2^ Heart Repair and Regeneration Laboratory, Department EMC, Faculty of Science and Medicine, University of Fribourg, Fribourg, Switzerland; ^3^ Department of Muscle and Bone Metabolism, Institute of Aerospace Medicine, German Aerospace Center, Cologne, Germany

**Keywords:** diabetes, exercise, genotype, signalling, angiotensin

## Abstract

Homozygous carriers of the deletion allele in the gene for angiotensin-converting enzyme (ACE-DD) demonstrate an elevated risk to develop inactivity-related type II diabetes and show an overshoot of blood glucose concentration with enduring exercise compared to insertion allele carriers. We hypothesized that ACE-DD genotypes exhibit a perturbed activity of signaling processes governing capillary-dependent glucose uptake in vastus lateralis muscle during exhaustive cycling exercise, which is associated with the aerobic fitness state. 27 healthy, male white Caucasian subjects (26.8 ± 1.1 years; BMI 23.6 +/− 0.6 kg m^−2^) were characterized for their aerobic fitness based on a threshold of 50 ml O_2_ min^−1^ kg^−1^ and the ACE-I/D genotype. Subjects completed a session of exhaustive one-legged exercise in the fasted state under concomitant measurement of cardiorespiratory function. Capillary blood and biopsies were collected before, and ½ and 8 h after exercise to quantify glucose and lipid metabolism-related compounds (lipoproteins, total cholesterol, ketones) in blood, the phosphorylation of 45 signaling proteins, muscle glycogen and capillaries. Effects of aerobic fitness, ACE-I/D genotype, and exercise were assessed with analysis of variance (ANOVA) under the hypothesis of a dominant effect of the insertion allele. Exertion with one-legged exercise manifested in a reduction of glycogen concentration ½ h after exercise (−0.046 mg glycogen mg^−1^ protein). Blood glucose concentration rose immediately after exercise in association with the ACE-I/D genotype (ACE-DD: +26%, ACE-ID/II: +6%) and independent of the fitness state (*p* = 0.452). Variability in total cholesterol was associated with exercise and fitness. In fit subjects, the phosphorylation levels of glucose uptake-regulating kinases [AKT-pT308 (+156%), SRC-pY419, p38α-pT180/T182, HCK-pY411], as well as cytokine/angiotensin 1-7 signaling factors [(STAT5A-pY694, STAT5B-pY699, FYN-pY420, EGFR-pY1086] were higher in angiotensin converting enzyme I-allele carriers than ACE-DD genotypes after exercise. Conversely, the AKT-S473 phosphorylation level (+117%) and angiotensin 2’s blood concentration (+191%) were higher in ACE-DD genotypes. AKT-S473 phosphorylation levels post-exercise correlated to anatomical parameters of muscle performance and metabolic parameters (*p* < 0.05 and │r│>0.70). The observations identify reciprocal alterations of S473 and T308 phosphorylation of AKT as gatekeeper of a post-translational dysregulation of transcapillary glucose uptake in ACE-DD genotypes which may be targeted in personalized approaches to mitigate type II diabetes.

## Introduction

Glucose metabolism is a main source of energy production in skeletal muscle during exercise. With the onset of exercise, there is a rapidly enhanced metabolic conversion of glucose in contracting skeletal muscle to yield mobile energy equivalents in the form of ATP (Hargreaves and Spriet 2020). The enhanced glucose use is fuelled by the degradation of glycogen stores in muscle fibers and the import of blood-borne glucose (Richter and Hargreaves 2013), which is supported by the hepatic release of glucose (Kjaer 1998; Fujimoto et al., 2003; Brooks 2020). Depending on the intensity of exercise, the rapid initial steps of glucose metabolism by glycolysis are coupled *via* the oxygen-dependent conversion of pyruvate in mitochondria to a highly efficient, but slower, production of ATP (Baker et al., 2010). Thereby, anaerobic and aerobic aspects of glucose metabolism influence the rate of energy delivery to fuel excitation-contraction coupling and consequently the fatigue resistance of power output in contracting skeletal muscle. At the whole-body level, the relative involvement of anaerobic and aerobic aspects of glucose metabolism is quantifiable by indirect calorimetry through measurable differences in the dissipated carbon dioxide relative to inhaled oxygen (Patel et al., 2022). Meanwhile, subjectively perceived exhaustion coincides with glycogen depletion at the local muscle level (Impey et al., 2018).

Glucose uptake in skeletal muscle is controlled dependent on capillary perfusion and the possibly interconnected transcapillary glucose transport *via* facilitative glucose-transporter 4 (GLUT-4; Richter and Hargreaves 2013). Both are regulated by endocrine and haemodynamic factors, of which insulin- and contraction-induced vasodilatation of arterioles are the main elements upregulating capillary perfusion (Steinberg et al., 1994). Such contraction-induced vasodilatation overrides angiotensin 2-related (inhibitory) processes (Korthuis 2011) that produce vasoconstriction and lower glucose-uptake *via* antagonistic actions on vasodilatative bradykinin/NO signaling in skeletal muscle (Henrickson and Jacob 2003); as pointed out by the pharmacological inhibition of angiotensin converting enzyme (ACE) that produces the octapeptide angiotensin 2 through cleavage of the precursor peptide, angiotensin 1. During physical exercise, the vasoconstrictor actions of angiotensin 2, whose blood concentration is increased with exercise intensity, are mitigated locally by vasodilatory actions of contraction (Clifford and Hellsten, 2004; Korthuis 2011). Skeletal muscle is probably the largest contributor to glucose homeostasis during exercise with contraction-induced glucose uptake (Surapongchai et al., 2018), suggesting a critical implication of angiotensin 2-related differences for glucose handling.

Homozygous carriers of the deletion allele (D) in the *ACE* gene (i.e., ACE-DD genotypes), that shows responsible for the production of angiotensin, typically demonstrate an enhanced potential for the production of angiotensin 2 compared to insertion allele (I) carriers of the ACE gene, i.e., ACE-ID and ACE-II genotypes (reviewed by Vaughan 2013; Vaughan et al., 2016). Intriguingly, angiotensin 2 levels and the relatively frequent ACE-I/D genotype have been associated with diabetes and insulin resistance (Nicola et al., 2001; Feng et al., 2002; Chu and Leung 2009). For instance, ACE-DD genotypes demonstrate an elevated risk of developing inactivity-related type II diabetes, which is characterized by a substantially elevated blood concentration of glucose in the fasted state at rest (Feng et al., 2002). Interestingly, we identified that ACE-DD genotypes demonstrate unusually high levels of blood glucose concentration compared to insertion allele carriers (ACE-ID/-II) with exhaustive endurance exercise to pre-diabetic levels (Valdivieso et al., 2017). The observed rise in blood glucose concentration was related to the failure of ACE-DD genotypes to elevate capillary perfusion with exhaustive exercise (van Ginkel et al., 2015a), and possibly lower capillary density in peripheral skeletal muscle (Vaughan et al., 2016). The formerly mentioned genotype differences in regulative and capacitive aspects of capillary supply in peripheral tissues, promote an excessive depletion of muscle glycogen, and citrate cycle-related muscle metabolites in ACE-DD genotypes, which depends on the individual aerobic fitness (Valdivieso et al., 2017).

There is a wealth of data on the preventive or delaying effect of physical activity against type II diabetes (Thomas et al., 2006; World Health Organization 2011; Smith et al., 2016), and a wealth of data is available on the molecular mechanism underpinning exercise- and insulin-induced glucose uptake and its relation to type II diabetes (Stanford and Goodear, 2014; Carson 2017). In the last 2 decades, there has been a gain in knowledge on the eminent role of angiotensin 2-modulated (glucose) metabolism in skeletal muscle and its association with diabetes (Nicola et al., 2001; Henriksen and Jacob 2003; Chu and Leung 2009; Korthuis 2011). The contribution of angiotensin to the observed variability in the glucose metabolism with exercise, and the role played by physical fitness-related differentiation of skeletal muscle composition (Dengel et al., 2002; Schüler et al., 2017; Brooks 2020), is only incompletely understood at the mechanistic level. The implicated molecular processes possibly involve altered intracellular angiotensin and insulin signaling, which may lower glucose uptake and expression of glucose metabolism associated gene alike reported for the pharmacological inhibition of ACE (Henriksen and Jacob 2003; Mathes et al., 2015). Intriguingly, variability in anatomical and functional aspects of aerobic glucose and lipid metabolism in contracting knee extensor muscle is associated with the ACE-I/D genotype in interaction with the aerobic fitness state (Vaughan et al., 2013; Valdivieso et al., 2017; Gasser et al., 2022). For instance, higher increments in blood glucose concentration, respiration exchange ratio and glycogen depletion have been reported for ACE-DD genotypes during exhaustive one-legged exercise of healthy individuals, when conversely a higher degree of muscle deoxygenation and higher volume densities of mitochondria and intra-myocellular lipids are detected in aerobically fit ACE I-allele carriers. The observed differences advocate that aerobically fit ACE I-allele carriers are endowed with an improved capacity for intramuscular import and aerobic metabolization of blood-borne glucose (and lipid) during exhaustive exercise compared to non-carriers of the ACE I-allele. These observations motivate the hypothesis that ACE-DD genotypes exhibit compared to ACE I-allele carriers a perturbed capacity to activate signaling processes governing capillary-dependent glucose uptake in contracting skeletal muscle during exhaustive cycling exercise, which is associated with the state of aerobic fitness. For testing this assumption, we monitored blood metabolites and muscle glycogen concentration and the phosphorylation levels of forty-five proteins of intracellular signaling before and after a one-legged bout of exhaustive exercise. We assessed whether observable differences in exercise-induced responses were associated with muscle capillarisation and the ACE-I/D genotype in healthy male subjects.

## Methods

### Subjects

Twenty-seven healthy, male white Caucasian subjects (26.8 ± 1.1 years; BMI:23.6 +/− 0.6 kg m^−2^) were recruited from White British men of the Greater Manchester Area. Exclusion criteria were smoking, long-term ill-health, an age below 18 years or over 40 years, and a relative VO_2_max below 40 ml O_2_ min^−1^ kg^−1^ or above 60 ml O_2_ min^−1^ kg^−1^ (as determined post-hoc). The Ethics committee of Manchester Metropolitan University specifically approved this study. The investigation was conducted according to the principles expressed in the Declaration of Helsinki and published guidelines (Ramos-Jimenez et al., 2008; Harriss and Atkinson 2011). Informed consent, written and oral, was obtained from the participants.

### Design

Subjects reported to the laboratory on two occasions to estimate aerobic capacity during one session of two-legged cycling exercise and one session of exhaustive one-legged cycling exercise in the fasted state in the morning to test the metabolic response. The two visits were separated by at least 2 days. Oxygen uptake, respiration exchange ratio (RER), and the blood concentrations of selected metabolites, i.e., glucose, triacylglycerol (TAG), total cholesterol (T Cholesterol), low- (LDL) and high-density lipoprotein (HDL), total ketone (ketones), were monitored in samples being collected prior to, and ½, 3, and 8 h after exercise as described (Valdivieso et al., 2017). Concomitantly, biopsies were collected from m. Vastus lateralis of the non-exercised leg immediately before exercise, and ½, 3, and 8 h after exercise from the exercising leg, respectively. Between the first and last biopsy subjects rested under supervision without physical activity reading a book, watching a movie, listening to music, or surfing the web, and consuming a meal based on sandwiches and water, if desired. The biopsy material was used to quantify muscle capillarisation, and the concentration of glycogen and specific phosphorylation of a panel of 45 proteins involved in key intracellular signaling processes was quantified with a phospho-kinase array (Proteome Profiler, R&D Systems). In the two-legged exercise test, the aerobic fitness state was determined post-hoc based on VO_2_max values below (unfit) or above (fit) 50 ml O_2_ min^−1^ kg^−1^, respectively. The ACE-I/D genotype was determined, in a double-blind manner, with allele specific polymerase chain reactions on genomic DNA being isolated from a buccal swap collected before the one-legged exercise. The MOS 36-Item Short-Form Health Survey (SF-36) and an activity questionnaire (Ware and Sherbourne 1992; Howley and Franks, 2003) were completed to assess the level of physical activity and the current medical health.

### Two-legged cycling exercise

Subjects carried out an incremental test of exercise to exhaustion on a stationary cycle ergometer (Ergometrics Ergoline 800, Jaeger, Bitz, Germany) in an air-conditioned room at 23°C with the concomitant assessment of cardiopulmonary parameters essentially as described (Vaughan et al., 2016). On this occasion age, body weight and height were measured, and the BMI was calculated during an initial visit. Then subjects completed a lifestyle questionnaire composed of 31 questions as modified from a previous short-form 36 (Ware and Sherbourne, 1992).

Cardiopulmonary measurements were carried out through a mouthpiece with a breath-by-breath technique using a stationary testing device (MetaLyser^®^ 3B, Cortex, Leipzig, Germany). The saddle length was adjusted to a position where the knee was extended at an approximately 175° angle when subjects were seated with the shoe heel placed on the pedal. The test started with 2 min of baseline recording followed by 3 min warm-up at 80 W and 80 rpm. The intensity was increased by 25 W every minute until exhaustion. An intensity level was considered achieved when 80 rpm were held for at least 50 s. Respiration was followed during a cool-down phase of 3 min at 80 W and 80 rpm followed by 2 min of rest. Test results were recorded at 3 s intervals with the MetaSoft^®^ software (Cortex, Leipzig, Germany) and analysed offline with the method “maximal oxygen uptake’ for absolute and body mass-related VO_2_max and RER following the exercise. VO_2_max, and the corresponding maximum aerobic power output (Pmax), were identified based on the criteria that VO_2_ reached a plateau of a steady maximal value under the imposed high workload, when RER was above 1.05, and before VO_2_ fell off because the pedal rate fell consistently below 70 rpm despite verbal encouragement (for a review see Vaughan et al., 2016). The VO_2_ values at the plateau varied within 1% of the average values and the plateau was maintained on average over 26 s VO_2_max was determined as the highest mean of VO_2_ values averaged over 30 s in the plateau phase. If a VO_2_ plateau did not manifest during the test, the ergospirometry was repeated on a subsequent day.

### One-legged cycling exercise

Subjects reported after an overnight fast and 2 days of reduced physical activity in the laboratory. A resting biopsy was collected under anaesthesia from the vastus lateralis muscle of the non-dominant leg. A 5-ml blood sample was drawn from the Cephalic vein into a tube sprayed with dry EDTA (K2E BD Vacutainer^®^, Belliver Industrial Estate, Plymouth, United Kingdom) and placed on ice. A 2-ml aliquot was rapidly processed to “quantify serum angiotensin 2 concentration” and determine the concentration of selected metabolites.

Subsequently, subjects completed a one-legged exercise test with the dominant leg on the stationary cycle ergometer (Ergometrics Ergoline 800, Jaeger, Bitz, Germany) at a performance-matched intensity and a set cadence of 80 rpm. During the exercise, the pedal for the non-dominant leg was taken off. Saddle length was set to the value used for the two-legged exercise. The shoe of the dominant leg was attached to the pedal with duct tape. The other leg rested on the frame in the middle of the ergometer. Subjects initially performed a warm-up at 15% of the predicted 2-legged Pmax, followed by 25 min of exercise at 30% of the 2-legged Pmax before the set intensity was ramped up in 10 W-increments per minute until exhaustion. A 3-min cool-down phase at 15% of the calculated 2-legged Pmax was allowed at the end of the exercise. VO_2_, VCO_2_ and ventilation were monitored with the MetaLyser^®^ 3B system (Cortex, Leipzig, Germany) and VO2max and maximal RER was determined.

### Genotyping

The buccal swap, collected with a cotton earbud, was frozen at −20°C in a sealed 15 ml tube (Sarstedt; Nümbrecht; Germany). DNA was extracted after thawing with 800 μl of methanol. The solution was air-dried, frozen overnight at −80°C, and resuspended in 100 μl of sterile water under heating to 65°C. DNA was recovered in the supernatant after a centrifugation step (5,000 g, 2 min, room temperature) and stored at −20°C. A second investigator blinded sample codes by sticking a label with a random, but unique, four-letter code on top. The code was handed to a third investigator unrelated to the study. Subsequently, the DNA samples were subjected with mock and camouflage samples to a polymerase chain reaction to type the ACE-I/D polymorphism according to the protocol described by Vaughan et al. (2013). The genotyping results were decoded through the involvement of the third investigator once the functional test and metabolic measures had been completed.

### Biopsy

An experienced physician collected muscle samples taken from the vastus lateralis muscle, at the point of maximal thickness. The overlying skin was shaved and sterilised (Videne Antiseptic Solution, Ecolab, Saint Paul, MN United States). A sterile drape from a wound care pack (Premier, Shermond Bunzel Retail & HealthCare Supplies Limited, Enfiled, Middlesex, United Kingdom) ensured sterile conditions. For local anaesthesia, 1 ml 2% Lidocaine was injected subcutaneously. Within 5 min, a 5-mm incision was made with a scalpel and a muscle sample was extracted and immediately processed by a skilled investigator. For the sample point prior to exercise, the sample was collected with a rongeur using the conchotome technique; while post-exercise samples were collected using a biopsy needle (TSK Acecut 11G, Emergo Europe, The Hague, Netherlands). Firm pressure was applied to the biopsy site until the bleeding stopped. The wound was then closed (Steri-Strip, 3M Health Care, Germany) and dressed (Mepore Ultra, Molnlycke Healthcare, Sweden). Subjects were discharged with a pressure bandage for the first 4 h after the biopsy sample to reduce further bleeding.

Biopsy samples were rapidly frozen in liquid nitrogen while shaking the sample. Pre-exercise samples were cut into two pieces before being frozen; one being mounted with Tissue-Tek^®^ O.C.T. TM Compound (Weckert Labortechnik, Kitzingen, Germany) on cork for histological analysis before freezing. Samples were stored airtight in a 2-ml tube (Eppendorf) until further processing.

### Measurements of muscle capillarisation

Capillaries were detected and analysed as described (van Ginkel et al., 2015b). In brief, pre-exercise biopsies were mounted, and 14-μm thick cryosections prepared at a cutting angle perpendicular to the major axis of muscle fibers. Capillaries were detected based on a lectin antibody, and the section was recorded at a ×10 magnification with an Axiocam MRC camera operated by an Axioskop 2 mot plus stage (Carl Zeiss, Oberkochen, Germany). Areas of the section corresponding to 0.15 mm^2^ where fibers were cut perpendicular and where no holes or other irregularities were present were selected. The areas were processed with the ImageJ software (version 1.6.0_33; http://imagej.nih.gov/ij) according to the published settings (van Ginkel et al., 2015b) to determine the number of capillaries per mm^2^ (capillary density) and the capillary-to-fiber ratio. Additionally, the mean cross-sectional area (MCSA) of slow (type I) and fast (type II) muscle fibers was determined on muscle cross-sections being stained with myosin isoform-specific antibodies as described (Vaughan et al., 2016). The values of at least 24 representative fibers per subject were analysed.

### Muscle glycogen

Cryosections (25 μm) were prepared from muscle biopsies and the section volume was estimated from microscopic measures of the cross-sectional area and the height of the sectioned tissue. An approximate of 1 mm^3^ tissue was homogenised in 100 μl of a PBS/inhibitor-cocktail [1 ml PBS +9 ml dH2O+ 1 complete Mini, EDTA-free tablet (Sigma Aldrich, Buchs, Switzerland) in a 1.5 ml Eppendorf tube by using a steel pistil (Behrens-Labortechnik, Germany). Total protein content was assessed in 3 μl homogenate against a BSA standard using the Pierce BCA Protein Assay Kit (Thermo SCIENTIFIC, Town, United States) and quantified at 562 nm on a 96-well plate with a Synergy HT spectrometer (BioTek Instruments Inc., Vermont United States). Glycogen was measured on 20 μl muscle homogenate against a glycogen standard with the Assay Kit (Abcam, Cambridge, United Kingdom). The reaction was developed at room temperature in a 96-well plate in the dark. The signal was detected at 564 nm using a Synergy HT spectrometer (BioTek, Lucerne, Switzerland). Glycogen levels were expressed relative to the total amount of muscle protein. The coefficient of variation for repeated measurements of the standard curve on different days was 3.1% for BSA-based measures of protein content and 0.1% for glycogen, respectively.

### Quantification of phosphorylation levels

45 proteins involved in intracellular signaling processes was quantified with a phospho-kinase array using the standard description (Proteome Profiler, R&D Systems). In brief, the soluble matter was extracted from muscle biopsies by grinding an approximate 10 mm^3^ biopsy material in 200 μl of ice-cold RIPA buffer (2% Triton X-100, 1% NP-40, 300 mM NaCl, 20 mM Tris base, 2 mM EDTA, 2 mM EGTA) supplemental protease inhibitor (complete Mini EDTA-free (Roche, Basel, Switzerland), and phosphatase inhibitors (Phosstop, Roche, Basel, Switzerland) with a Polytron mixer (PT1200E, Kinematica, Lucerne, Switzerland). Soluble protein was recovered from the supernatant of a centrifugation step at (5,000 g, 2 min, 4°C) and the corresponding protein content was determined with the Pierce^®^ BCA protein assay kit (Thermo Fischer Scientific, United States). 600 μg of soluble protein in (from two biological replicas per time point) were applied per pair of filter array, including two detection spots per feature. Signal detection was carried out with enhanced chemoluminescence and signal was recorded after an exposure of 1 and 30 min duration with a Fusion Solo S Edge Chemoluminescence imaging system (Vilber, Marne-la-Vallée, France). Signals were quantified with Quantity One software (Biorad, Cressier, Switzerland). The signal was corrected for the general background and averaged for the signals from the two corresponding spots per array. The resulting values of signal intensity (INT) in counts per standardized area (mm^2^) were related to the respective reference spots on the membrane. All spots were detected. The CV of repeated measures was 6%.

### COX4I1 transcript expression

Total RNA was isolated from muscle biopsies using the RNeasy Mini Kit (Qiagen) and 600 ng was reverse transcribed using the Omniscript RT Kit (Qiagen, Hombrechtikon, Switzerland) using random hexamers (Qiagen, Hombrechtikon, Switzerland). Subsequently, RT-qPCR was performed on ∼5 and 0.5 ng cDNA with specific primers to detect the marker of aerobic capacity in vastus lateralis muscle, i.e., level of mitochondrial gene transcript cytochrome c oxidase subunit IV isoform 1 (COX4I1; forward, 5′-GCC​ATG​TTC​TTC​ATC​GGT​TTC-3′; reverse, 5′-GGC​CGT​ACA​CAT​AGT​GCT​TCT​G-3′), and 28S rRNA (forward, 5′-ATA​TCC​GCA​GCA​GGT​CTC​CAA-3′; reverse, 5′-GAGCCA ATCCTTATCCCGAAG-3′) essentially as described (Desplanches et al., 2014). Reactions were run in duplicate using the KAPA SYBR FAST universal Kit (Labgene Scientific, Châtel St. Denis, Switzerland) according to manufacturer’s instructions. Relative transcript amounts were calculated using the comparative CT method, taking the efficiency of amplification for each template into account using the comparative method for the threshold cycle for target amplification as described (Schmutz et al., 2006). For each sample, transcript signals were standardized to 28S rRNA.

### Quantification of serum angiotensin 2 concentration

Angiotensin 2 levels were quantified with a validated commercial angiotensin 2 enzyme-linked immunoabsorbent assay (ELISA, SPIBio Bertin Pharma, Montigny le Bretonneux, France) from blood being collected from the cubital vene before and immediately after ramp exercise and processes essentially as described (van Ginkel et al., 2015a). In brief, the samples were immediately processed by centrifugation at 4°C (3,000 g for 12 min) in the presence of inhibitor cocktail (comprising O-Phenanthroline, Pepstatine A and ethylene-diamine-tetraacetic acid and polyhexamethylene biguanide). The supernatant plasma was concentrated *via* an C18 phenyl cartridge before ELISA based measurements were conducted vs. an internal standard.

### Blood serum metabolites

30 μl of capillary blood was used to measure the main metabolic substrates (glucose, triacylglycerol, total cholesterol, low- and high-density lipoprotein, and/or total ketones) using a portable whole blood test system (CardioCheck^®^, Polymer Technology Systems; Indianapolis, IN, United States). Glucose concentration was measured in the first minute after collection. The serum concentration of low-density lipoprotein and very-low-density lipoprotein cholesterol was calculated as described (Rifai et al., 1992). The coefficient of variation for repeated measurements was 2.6% for glucose and 3.9% for triglycerides, respectively.

### Statistics

Data were assembled and displayed as Box-Whisker plots using MS-Excel (Microsoft Office Professional Plus 2016; Kildare, Ireland) and exported into the Statistical Package for the Social Sciences (SPSS version 23, IBM, Armonk, United States). Analysis of variance (ANOVA) with post hoc test for the least significant difference was used to assess interaction effects of aerobic fitness [unfit, fit] x the ACE-I/D genotype [I-allele, no I-allele] under the hypothesis of a dominant effect of the insertion allele. Where applicable, effects of exercise, and interactions with ACE-I/D genotype x aerobic fitness were assessed with a repeated-measures ANOVA. Effects of time [½, 8 h post exercise] and interactions with the ACE-I/D genotype [I-allele (ACE-ID/II), no I-allele (ACE-DD)] and factor identity [phosphorylation of 45 signaling proteins] were assessed with a multivariate ANOVA.

Data for each parameter were assessed by a Kolmogorov-Smirnov test and inferential statistics based on a Levene and/or Mauchly test to verify whether the data meet the assumption of normality and equality of variance, respectively. In case the assumption was violated we subjected the data to the non-parametric Kruskal–Wallis test, where applicable. Effects were declared significant at *p* < 0.05. The strength of linear relationships between phosphorylation levels and anatomical/physiological parameters was analysed with a two-step procedure. First, Pearson product-moment correlations were carried out for the values from the repeated sample points, 30- and 480-min post exercise and average r- and *p*-values calculated. Subsequently, linear relationships which met the criteria of |r|>0.7 and *p* < 0.10 were singly verified for the significance of repeated measures correlations (rrm) using an online available algorithm under a threshold for significance of |rrm|>0.7 and *p* < 0.05 (https://lmarusich.shinyapps.io/shiny_rmcorr/; Marusich and Bakdash 2021). Average values are given as mean ± standard deviation (SD), and where applicable minima and maxima are given in brackets.

## Results

### Subject characteristics

Table 1 summarizes the average physiological characteristics of the studied twenty-seven male subjects. Based on the introduced threshold of 50 ml O_2_ min^−1^ kg^−1^, nine and eighteen subjects qualified as aerobically fit or unfit, respectively. The aerobically fit individuals performed on average at least 6 h of physical activity per week at an intensity that required an extra cardiovascular effort. On average the aerobically fit subjects demonstrated superior values for maximal oxygen uptake (+24%) and power output (29%) during the two-legged cycling exercise to exhaustion (Table 1). Maximal oxygen uptake (+15%) and power output (19%) one-legged cycling exercise demonstrated trends for higher values in the aerobically fit subjects. Three subjects of a total of thirteen I-allele carriers, were identified as to represent homozygous I-allele carriers.

**TABLE 1:** Physiological characteristics.

	N	Age	Body mass	Height	BMI	Pmax 2-leg	VO_2_max	RER rest	RER max	Pmax	VO_2_max
		[years]	[kg]	[cm]	[kg m^−2^]	[Watt]	[mL O_2_ min-1]			[Watt]	[mL O_2_ min-1]
					2-leg	2-leg	2-leg	2-leg	1-leg	1-leg	
All	27	26.8 ± 5.7	77.9 ± 10.4	181.6 ± 6.8	23.6 ± 3.1	318.4 ± 57.7	4,142.4 ± 697.8	0.74 ± 0.05	1.08 ± 0.05	189.6 ± 43.6	3,438.2 ± 646.9
unfit	9	24.8 ± 5.1	74.8 ± 11.7	179.4 ± 7.5	23.3 ± 4.2	266.7 ± 33.9	3,563.8 ± 449.1	0.77 ± 0.06	1.10 ± 0.06	168.3 ± 24.9	3,128.3 ± 530.7
Fit	18	27.7 ± 5.9	79.3 ± 9.8	182.6 ± 5.9	23.8 ± 2.5	342.9 ± 50.9	4,416.4 ± 632.2	0.71 ± 0.08	1.07 ± 0.08	200.3 ± 47.5	3,593.1 ± 656.8
p fitness		0.291	0.479	0.322	0.907	**0.002**	**0.003**	0.078	0.375	0.098	0.082
ACE-DD	14	25.4 ± 5.2	75.8 ± 10.1	181.9 ± 7.9	22.9 ± 2.6	305.7 ± 55.8	4,126.5 ± 761.8	0.76 ± 0.07	1.09 ± 0.07	189.6 ± 40.8	3,423.8 ± 673.9
ACE-ID/II	13	28.6 ± 6.1	80.3 ± 10.8	181.5 ± 5.8	24.4 ± 3.6	328.5 ± 63.1	4,136.9 ± 797.8	0.72 ± 0.07	1.07 ± 0.07	187.3 ± 50.1	3,450.2 ± 695.9
p genotype		0.437	0.326	0.855	0.319	0.462	0.912	0.366	0.609	0.875	0.907
p genotype x fitness		0.399	0.636	0.136	0.709	0.364	0.172	0.519	0.877	0.281	0.219

*Mean ± SD for the anthropometric and physiological characteristics of the studied 27 subjects as assessed at rest, during one-legged cycling exercise in the morning after an over-night fast, and two-legged cycling exercise on an ergometer. Bold values indicate effects which were deemed significant at p < 0.05. ANOVA for the effects of the ACE I-allele (i.e., genotype) × aerobic fitness (fitness) with post-hoc test of least significant difference.*

### Metabolic characteristics of the exhaustive one-legged exercise

**FIGURE 1:**
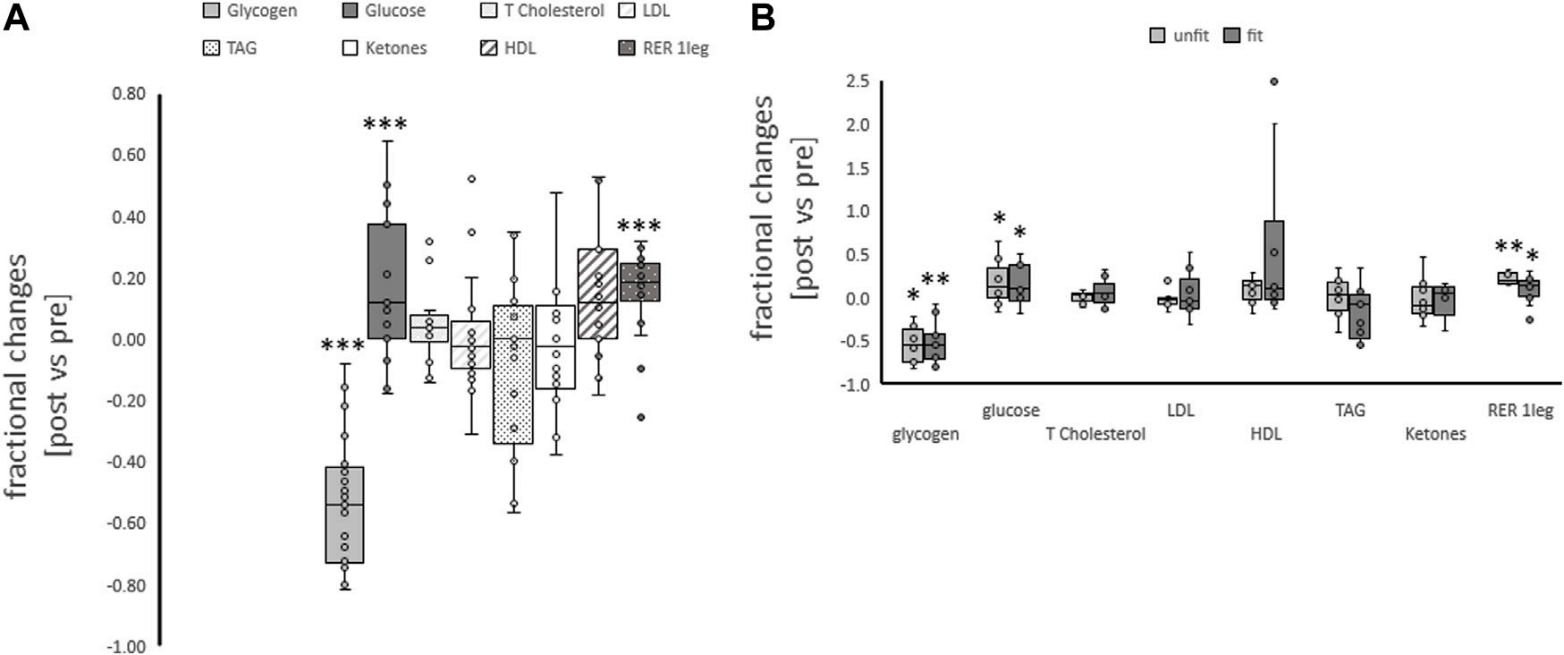
*Metabolic changes with one-legged exercise.* Box-Whisker plots (box: first and third quartile, whisker minima and maxima, line: median, x: mean, circles: non overlapping data points) for the fractional changes for blood metabolites and respiration exchange ratio immediately post one-legged exercise, and muscle glycogen concentration 30 min post one-legged exercise combined **(A)** and split for aerobic fitness state **(B)**. *, **, ***p < 0.05, <0.01, <0.005, respectively, for significant post vs. pre differences. ANOVA for the repeated factor of exercise and the factor of aerobic fitness. N = 27.

At rest, after on overnight fast the respiration exchange ratio was by a value of 0.14 higher in the fit compared to unfit subjects (Table 2). Subjects exercised with the dominant leg up to an imposed average intensity of 190 W during a ramp to exhaustion that followed 25-min of exercise at a steady load of 30% of the 2-legged Pmax. Such one-legged exercise increased the respiration exchange ratio from average values of 0.81 ± 0.03 to 0.91 ± 0.01 (Table 1; Figure 1A). Glycogen concentration per muscle protein in *m. Vastus lateralis* was reduced (in all subjects) by an average of 0.046 mg mg^−1^ protein (Table 2; Figure 1A). Concomitantly the blood concentration of glucose was increased by 0.74 mM, while the levels of other metabolic factors were maintained (Table 2).

**TABLE 2:** Metabolic characteristics.

	n	Glucose		Total cholesterol	Low density lipoprotein	Triacylglycerol	Ketones	[mM]	High density lipoprotein	Muscle glycogen	RER	[mM]		[mg mg^−1^]
		[mM]		[mM]		[mM]				[mM]				
		pre	post	pre	post	pre	post	pre	post	pre	post	pre	post	pre	post	pre	max
all	27	4.12 ± 0.62	4.86 ± 1.20	3.79 ± 0.57	3.93 ± 0.73	2.46 ± 0.52	2.47 ± 0.78	1.00 ± 0.62	0.90 ± 0.57	0.58 ± 0.26	0.53 ± 0.21	0.96 ± 0.31	1.16 ± 0.42	0.078 ± 0.052	0.032 ± 0.021	0.81 ± 0.16	0.91 ± 0.05
p exercise		**0.016**		0.546		0.982		0.362		0.160		0.126		**<0.001**		**<0.001**	
unfit	9	4.11 ± 0.81	4.76 ± 1.23	3.49 ± 0.27	3.55 ± 0.33	2.34 ± 0.30	2.29 ± 0.27	1.00 ± 0.69	0.99 ± 0.75	0.55 ± 0.24	0.50 ± 0.15	0.95 ± 0.30	1.03 ± 0.30	0.091 ± 0.066	0.033 ± 0.012	0.72 ± 0.03	0.88 ± 0.03
fit	18	4.17 ± 0.51	4.86 ± 1.15	3.98 ± 0.68	4.18 ± 0.85	2.53 ± 0.64	2.60 ± 0.98	0.95 ± 0.51	0.85 ± 0.38	0.62 ± 0.25	0.57 ± 0.30	0.97 ± 0.30	1.22 ± 0.42	0.075 ± 0.042	0.033 ± 0.025	0.86 ± 0.17	0.93 ± 0.08
p fitness (pre)		0.867		**0.039**		0.599		0.969		0.901		0.457		0.452		**0.025**	
p genotype (pre)		0.681		0.669		0.941		0.418		0.129		0.094		0.450		0.653	
p fitness		0.452		**0.003**		0.260		0.556 ^a^		0.901		0.418 ^a^		0.472		**0.006**	
p fitness x exercise		0.576		0.678		0.627		0.381		0.370		0.457		0.489		0.130	
ACE-DD	14	4.17 ± 0.79	5.27 ± 1.23	3.76 ± 0.67	3.89 ± 0.60	2.44 ± 0.64	2.43 ± 0.75	0.84 ± 0.41	0.83 ± 0.37	0.51 ± 0.30	0.46 ± 0.19	1.05 ± 0.41	1.18 ± 0.52	0.086 ± 0.060	0.033 ± 0.011	0.77 ± 0.11	0.88 ± 0.07
ACE-ID/II	13	4.07 ± 0.50	4.30 ± 0.65	3.82 ± 0.54	3.96 ± 0.87	2.47 ± 0.47	2.50 ± 0.79	1.15 ± 0.72	0.96 ± 0.69	0.65 ± 0.18	0.59 ± 0.22	0.88 ± 0.18	1.14 ± 0.38	0.070 ± 0.043	0.031 ± 0.025	0.84 ± 0.14	0.93 ± 0.04
p genotype		**0.020**		0.519		0.811		0.820 ^a^		0.129		0.368 ^a^		0.399		0.284	
p genotype x exercise		0.060		0.911		0.741		0.765		0.610		0.758		0.565		0.804	
p genotype x fitness		0.902		0.948		0.981		0.056		0.459		0.551		0.429		0.993	
p genotype x fitness x exercise		0.924		0.897		0.793		0.792		0.441		0.859		0.646		0.667	

*Mean ± SD for muscle glycogen and assessed metabolic characteristics in whole blood of the studied 27 subjects as assessed before and after exhaustive one-legged cycling exercise in the morning after an over-night fast. Bold values indicate effects which were deemed significant at p < 0.05. Repeated ANOVA for the effects of exercise x ACE I-allele (i.e., genotype) x aerobic fitness (fitness) with post-hoc test of least significant difference.*

a *Kruskall Wallis Test.*

### Fitness-associated variability in metabolic parameters

The concentration of neither of the assessed metabolic parameters altered with one-legged exercise in association with the fitness state (Table 2; Figure 1B). The fractional changes for the respiration exchange ratio demonstrated a trend (*p* = 0.074) for a higher increase in the unfit than fit subjects.

### Association of metabolic changes during one-legged exercise to exhaustion with the ACE-I/D genotype

**FIGURE 2:**
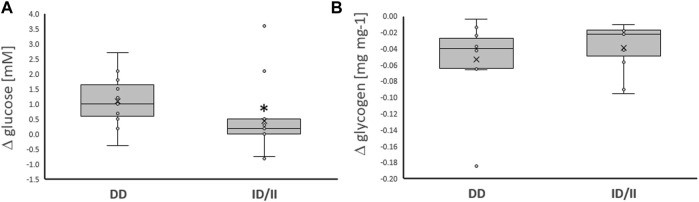
*Exercise-induced alterations of glucose metabolism related compounds per genotype.* Box-Whisker plots of the differences in the concentration of blood glucose **(A)** and muscle glycogen **(B)** immediately and 30 min, respectively, after one-legged exercise in carriers (ACE-ID/II) and non-carriers (ACE-DD) of the ACE I-allele. *, *p* < 0.05 for post vs. pre differences between genotypes. ANOVA for the factor ACE-I/D genotype. N = 27.

**FIGURE 3:**
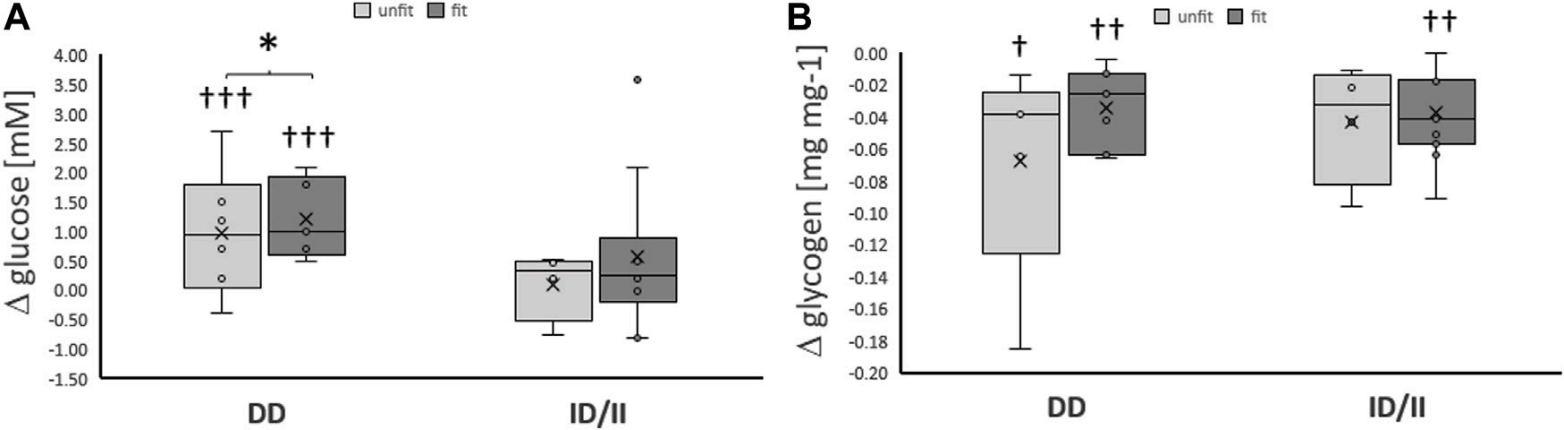
*Exercise-induced alterations of glucose metabolism related compounds per genotype and fitness state.* Box-Whisker plots of the differences in the concentration of blood glucose **(A)** and muscle glycogen **(B)** immediately and ½ hour, respectively, after one-legged exercise in association with the ACE I-allele (i.e., genotype) and fitness state. ^†, ††, †††^ *p* < 0.05, <0.01 < 0.001 for exercise-induced changes. *, *p* < 0.05 for differences between genotypes. Repeated measures ANOVA for the factor ACE-I/D genotype x exercise. N = 27.

Variability in exercise-induced alterations of blood glucose concentration, was associated with the ACE-I/D genotype (Table 2; Figure 2A). The effect resolved in an exaggerated increase in the concentration of blood glucose in ACE-DD genotypes (+26%) compared to I-allele carriers (7%) immediately after one-legged exercise, which did not depend on the fitness state (Figure 3A). Other metabolic parameters, including glycogen depletion (Figure 2B), did not demonstrate ACE-I/D genotype associated alterations with one-legged exercise (Supplementary Figure S1), not further interactions with the fitness state (Figure 3B; Table 2).

### ACE-I/D genotype effects on muscle signaling after exercise

For the aerobically fit subjects before ramp exercise, angiotensin 2 concentration in blood serum was higher in ACE-DD genotypes than in I-allele carriers (from 27.1 ± 4.1 vs. 9.3 ± 2.1 pg ml^−1^, *p* = 0.012). Angiotensin-2 concentration was increased immediately post-exercise (15.2 ± 4.1 to 36.3 ± 17.0 pg ml^−1^) and this did not differ between carriers and non-carriers of the ACE I-allele (*p* = 0.316).

**FIGURE 4:**
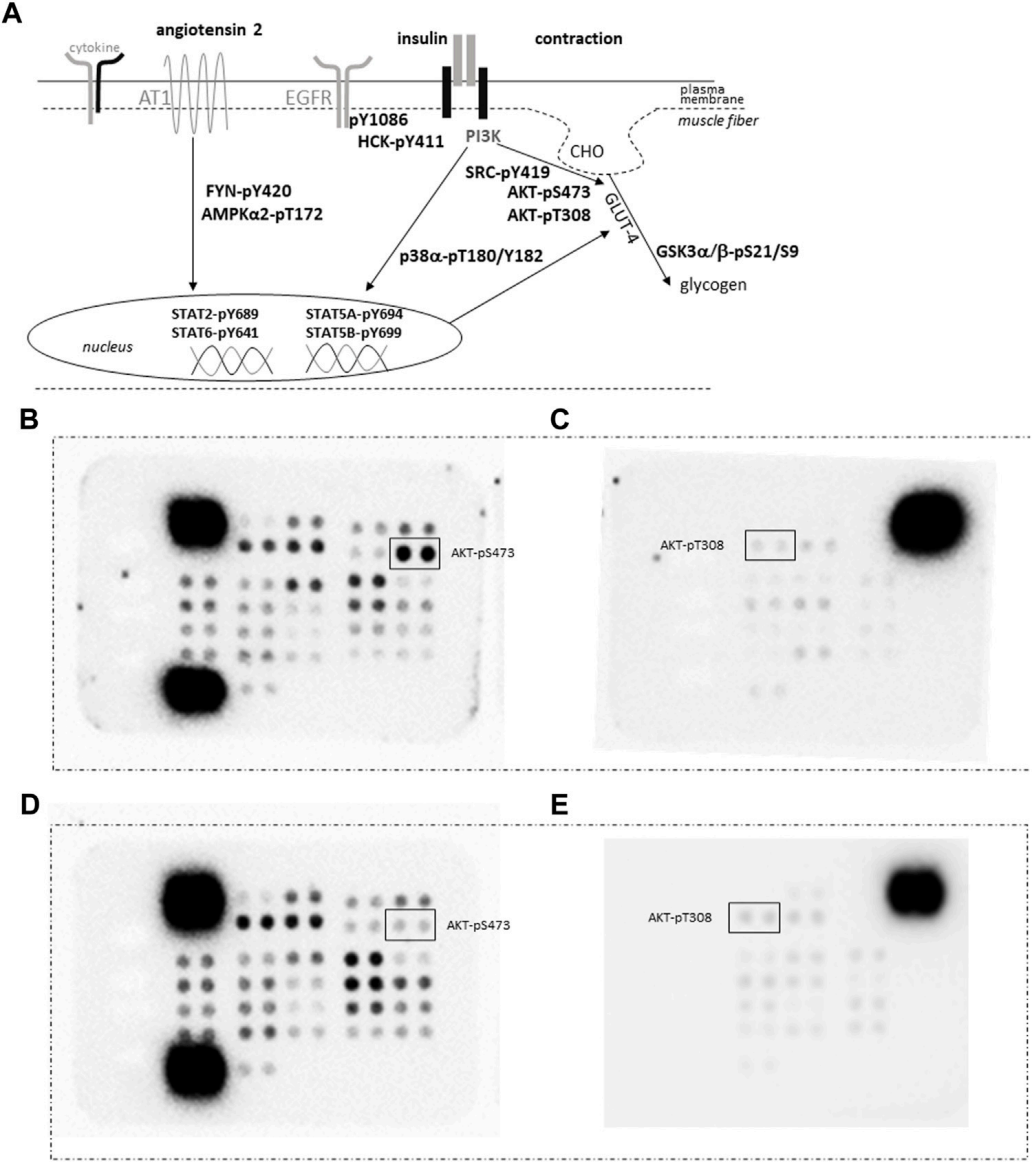
*Quantification of protein phosphorylation.* **(A)** Sketch of the assessed phosphorylation of signaling proteins being involved in GLUT-4 mediated glucose import as induced by contraction and insulin, angiotensin and cytokine signaling. Abbreviation: AT1, angiotensin receptor 1; CHO, glucose. **(B–E)** Images depicting the detection of the phosphorylation content for vastus lateralis muscle samples from an ACE-DD **(B,C)** and ACE-II **(D,E)** genotype ½ hrs post exercise in pairs of filters of the phospho-kinase arrays. Each phosphorylation content is assessed as measurement from a pair of spots. The position of the spot pairs for AKT-pS473 and AKT-pT308 is indicated. Strongest signals correspond to the reference signals used to ‘standardize’ the assessed counts.

The phosphorylation level of 45 proteins involved in intracellular signaling processes was quantified with a phospho-kinase array (Figure 4). The phosphorylation levels of assessed signaling factors demonstrated main effects of the ACE-I/D genotype (*p* = 4.3 × 10–3) and factor identity (*p* = 4.0 × 10–67) and both interacted (*p* = 2.3 × 10–4). Time (*p* = 0.212), the interaction between time x factor identity (*p* = 0.993) did not demonstrate an effect on phosphorylation levels. There was an interaction between time × I-allele (*p* = 0.037). In carriers compared to non-carriers of the I-allele, the overall levels of protein phosphorylation were higher 0.5 h post exercise (+0.0050 ± 0.0009 INT * mm^2^, *p* = 9.6 × 10–7), and this trend continued 8 h post exercise (+0.0002 ± 0.0010 INT * mm^2^, *p* = 0.075). For the ACE I-allele carriers, the overall phosphorylation levels of assessed signaling factors were lower 8 h compared to 0.5 h post exercise (−0.0020 ± 0.0007 INT * mm^2^, *p* = 0.002), when no difference existed between the two time points for the non-carriers of the I-allele (*p* = 0.622). No interaction existed between time x factor identity x ACE-I/D genotype (*p* = 0.980).

**FIGURE 5:**
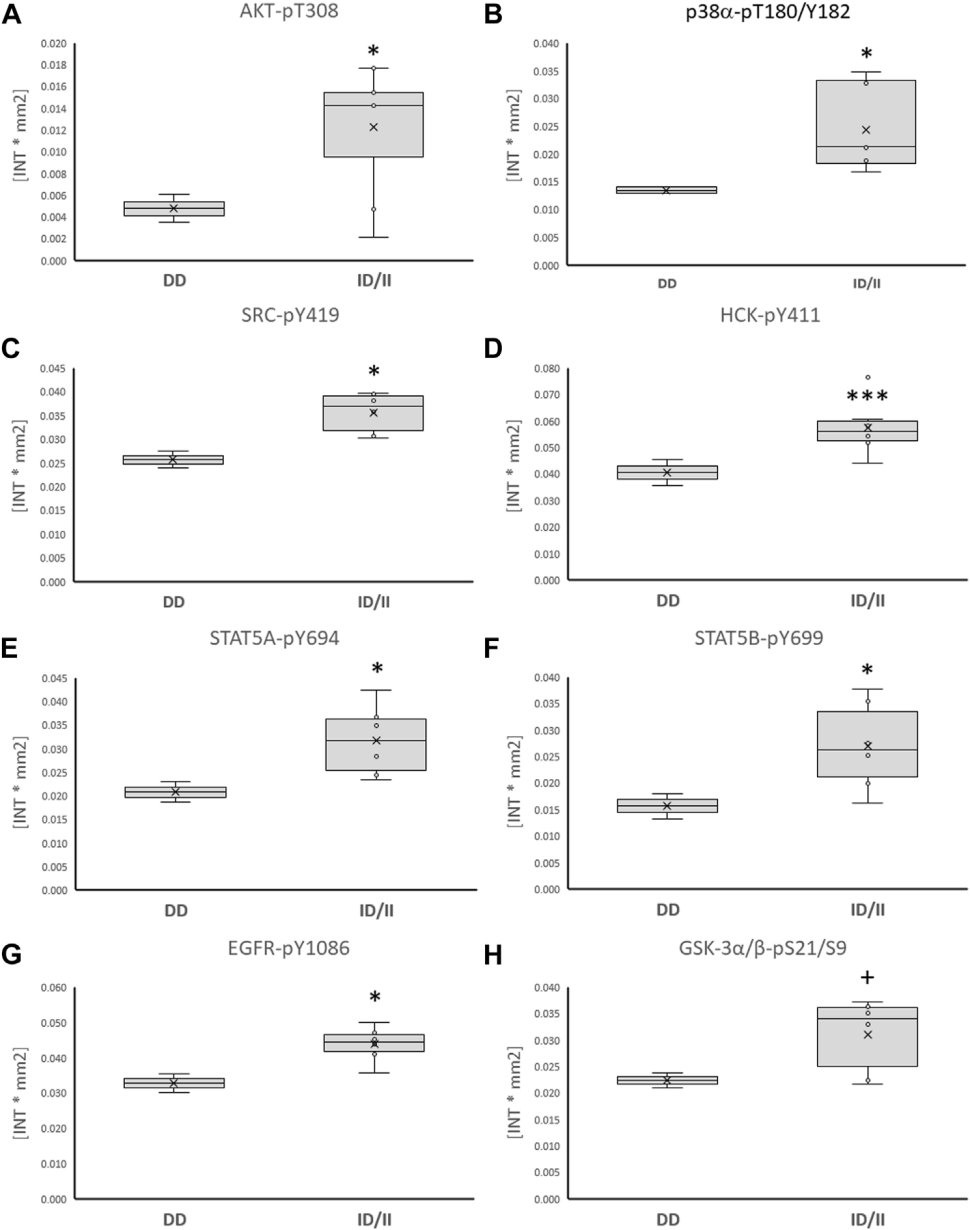
*Phosphotransferases demonstrating ACE-I/D genotype differences post exercise.* Box-Whisker plots of the (referenced) values for the phosphorylation of glucose metabolism related phosphotransferases in the fit carriers (ACE-ID/II) and non-carriers of the ACE I-allele (ACE-DD) as average of values ½ and 8 h after one-legged exercise. **(A)** AKT-pT308, **(B)** p38α-pT180/Y182, **(C)** SRC-pY419, **(D)** HCK-pY411, **(E)** STAT5A-pY694, **(F)** STAT5B-pY699, **(G)** EGFR-pY1086, **(H)** GSK3α/β-pS21/S9. +, * and ***, *p* < 0.10, <0.05, <0.001 for differences vs. ACE-DD genotype. ANOVA for the factor ACE-I/D genotype.

**FIGURE 6:**
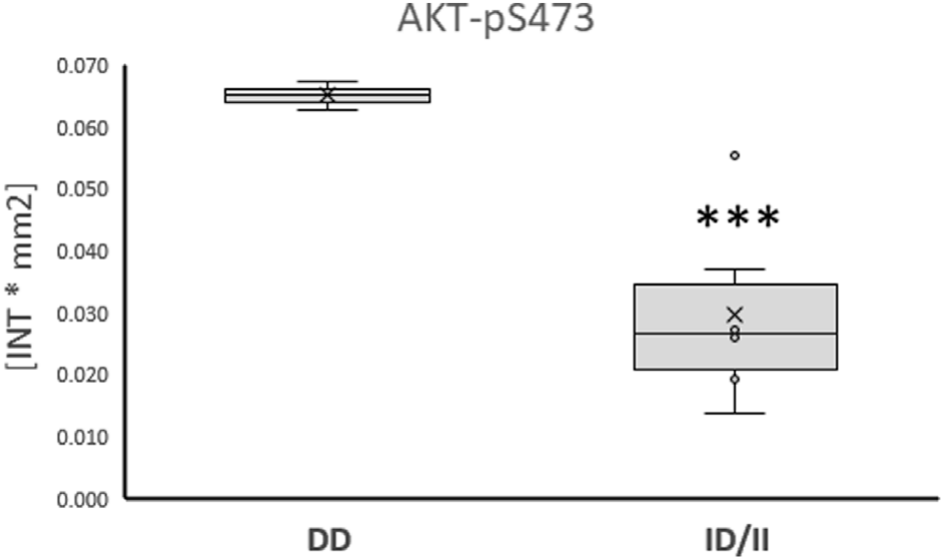
*Phosphotransferase demonstrating ACE-I/D genotype related ‘counter-regulation’ post exercise.* Box-Whisker plots of the (referenced) values for the phosphorylation of glucose metabolism related AKT-pS473 in the fit carriers (ACE-ID/II) and non-carriers of the ACE I-allele (ACE-DD) ½ - 8 h after one-legged exercise. ***, *p* < 0.001 for differences vs. ACE-DD genotype. ANOVA for the factor ACE-I/D genotype.

Subsequently, phosphorylation levels were analysed combined for the ½ and 8 h post exercise time points to isolate signaling factors which variable phosphorylation levels explained the interaction between ACE-I/D genotype and factor identity. For the aerobically fit subjects, the phosphorylation levels of glucose uptake-related kinases [AKT-pT308 (+156%), SRC-pY419 (+39%), p38α-pT180/T182 (+80%), HCK-pY411 (+42%)] were increased in carriers compared to non-carriers of the I-allele (i.e., ACE-II/ID vs. ACE-DD) between ½ to 8 h after exhaustive one-legged exercise (Figures 5A–D, G). As well, the phosphorylation of angiotensin 2/cytokine [(STAT5A-pY694 (+53%), STAT5B-pY699 (+72%), and angiotensin 1–7 [EGFR-pY1086 (+33%)] signaling factors was increased in ACE-II/ID vs. ACE-DD post exercise (Figures 5E–G). Concomitantly, the AKT-S473 phosphorylation level was selectively reduced in carriers compared to non-carriers of the ACE I-allele (−54%; Figure 6). The phosphorylation of the angiotensin 2-signaling-related PLC-γ1-pY783 was not affected ½ and 8 h after exercise (*p* = 0.43 and 0.61).

### Associations of muscle composition and metabolites at rest with fitness x ACE-I/D genotype

We assessed whether anatomy-related differences existed in skeletal muscle before one-legged exercise would be associated with the ACE I-allele and relate to metabolic efficiency. At rest, variability in MCSA of type I muscle fibers and the transcript level of the mitochondrial marker, COX4I1, in *m. Vastus lateralis* was associated with aerobic fitness, but not the ACE-I/D genotype (Table 3); both being higher in the aerobically fit than unfit subjects. MCSA of type II muscle fibers was associated with the ACE-I/D genotype, being larger in I-allele carriers than non-carriers (i.e., ACE-ID/II vs. ACE-DD genotype, Table 3).

**TABLE 3:** Muscle characteristics.

	n	MCSA type I	MCSA type II	type I area	capillary density	capillary to-fiber ratio	COX4I1
		[μm^2^]	[μm^2^]	[%]	[mm^−2^]		[/28S rRNA]
all	27	5,598.3 ± 2023.9	6,935.6 ± 2,713.9	36.6 ± 10.4	290.3 ± 41.6	2.22 ± 0.62	11.6 ± 16.1
unfit	9	4,424.6 ± 916.5	5,709.7 ± 1800.9	34.5 ± 11.1	283.7 ± 46.5	1.95 ± 0.54	5.2 ± 4.2
fit	18	6,451.9 ± 2,126.4	7,827.1 ± 2,926.6	38.2 ± 9.8	293.4 ± 39.9	2.34 ± 0.64	16.8 ± 21.2
p fitness		**0.006**	0.086	0.039	0.945	0.305	0.023
ACE-DD		4,863.0 ± 1,303.6	5,704.7 ± 1891.0	38.0 ± 7.1	281.4 ± 40.0	1.99 ± 0.49	10.0 ± 19.1
ACE-ID/II		6,415.4 ± 2,310.1	8,303.2 ± 2,629.9	35.2 ± 14.1	299.1 ± 42.2	2.44 ± 0.65	13.8 ± 11.5
p genotype		0.079	**0.019**	0.340	0.121	0.094	0.094
fitness x genotype		0.125	0.463	0.402	0.109	0.642	0.374

Mean ± SD for selected characteristics of the studied 27 subjects in *vastus lateralis* muscle before one-legged exercise and glycogen concentration with exercise. Bold values indicate effects which were deemed significant at *p* < 0.05. ANOVA for the effects of ACE I-allele (i.e., genotype) x aerobic fitness (fitness) with post-hoc test of least significant difference.

Variability in muscle capillarisation, i.e., capillary density and muscle-to-fiber ratio, and muscle glycogen concentration, before the one-legged exercise, was not associated with the fitness states, nor the interaction between the fitness state and ACE-I/D genotype (Tables 2, 3). Aerobically fit subjects demonstrated a 14% higher blood concentration total cholesterol (Table 2).

### Inter-relationships

96 largely significant correlations, meeting │r│>0.70 and *p* < 0.05, were identified between physiological parameters and the estimated levels of ACE-I/D genotype-dependent protein phosphorylation (Supplementary Table S1).

The phosphorylation level of AKT-S473 in m. Vastus lateralis muscle post-exercise, i.e., AKT-pS473, demonstrated the highest number of correlations with anatomical and metabolic parameters of muscle performance, i.e., MCSA of type I and type II muscle fibers, capillary-to-fiber ratio and fold changes in RER, and the blood concentrations of glucose and HDL with one-legged exercise (Table 5). As well, EGFRY1086 phosphorylation demonstrated correlations with exercise-induced fold changes in the blood concentrations of glucose and HDL, and RER.

The phosphorylation level of further signaling proteins correlated to physiological parameters pre- or post-exercise and fold changes in the blood concentration of lipid metabolism-related compounds (Supplementary Table S1). None of the assessed parameters was correlated with the concentration changes of muscle glycogen.

## Discussion

The provision and aerobic combustion of organic substrates, notably glucose, is a critical metabolic factor for the fueling of muscle contraction during intense forms of physical work for prolonged duration (Romijn et al., 1993; van Loon et al., 2001). Here we assessed the contribution of a frequent variation in the gene for a major regulator of capillary perfusion, i.e., the insertion/deletion gene polymorphism of angiotensin-converting enzyme to the observable variability in glucose metabolism-related parameters with one-legged type of cycling exercise to exhaustion, whether this would interact with the state of aerobic fitness, and whether this would be related to ACE-I/D genotype-associated differences in the phosphorylation of signaling factors that regulate glucose uptake in skeletal muscle fibers. We identified that the alteration of blood glucose concentration with exercise varies with the ACE-I/D genotype but is uncoupled from aerobic fitness. Conversely, variability in the muscle store of glucose, i.e., glycogen, with and without exercise was not associated with the interaction between the ACE-I-allele and fitness state. Notably, exercise-induced changes in indices of transcapillary uptake and combustion of glucose, and one lipid compound, in the exercised m. Vastus lateralis (i.e., concentration of blood glucose, RER, and capillary-to-fiber ratio, HDL) stood in a linear relationship with the phosphorylation level of AKT-S473 in exercised vastus that showed ACE-I/D associated differences in phosphorylation. Conversely, the blood concentration of the lipid-related compound, total cholesterol, demonstrated an exercise and aerobic fitness-associated variability, which was independent of the ACE-I/D genotype (Table 2). Notably, these effects were observed in the absence of any significant ACE-I/D-related differences in capillarisation. Collectively the findings implicate an important role of ACE-I/D associated signaling processes for variability in capillary-mediated glucose import into muscle fibers during exhaustive muscle work and implicate that this is related to acute, inter-individual differences in angiotensin-related AKT signaling post-exercise (Powers et al., 2018), which may be independent of capillary blood supply per se.

In respect to ACE-I/D genotype associated variability in exercise-induced alterations in blood glucose concentration, the inverse genotype dependence of T308 and S473 phosphorylation of AKT in m. Vastus lateralis post-exercise is intriguing. Only angiotensin 1–7 is known to increase the phosphorylation of both sites on AKT, while angiotensin 2 may decrease AKT phosphorylation in skeletal muscle (Morales et al., 2016; Powers et al., 2018). Our observation that only T308 phosphorylation of AKT was lower in ACE-DD genotypes with a higher blood concentration of angiotensin 2 prior to exercise, but when S473 phosphorylation was higher, calls for importance of considering the ACE-I/D genotype distribution, blood concentrations of angiotensin 2, and the specific occupancy of phosphorylation sites on AKT to render interpretations of exercise-induced AKT signaling in skeletal muscle meaningful for each individual.

The overshoot of blood glucose concentration with exhaustive exercise in the fasted state for ACE-DD genotypes in the studied white Caucasian men compares to the elevated susceptibility of the homologous genotype in populations from Northern China to demonstrate substantially elevated fasting blood glucose concentrations in the absence of exercise (Feng et al., 2002). This metabolic peculiarity reflects the incapacity of the body to control blood glucose concentration. It is typically due to peripheral insulin resistance associated with chronic physical inactivity World Health Organization, 2011; Surapongchai et al., 2018). Intriguingly, however, the ACE-I/D genotype-associated effect of exercise on blood glucose concentration in the current investigation did not depend on aerobic fitness which is normally thought to improve glucose handling (*p* = 0.945; Table 2; Supplementary Figure S1; reviewed in Álvarez et al. (2017). However, in the in-here studied population of healthy subjects, the ACE-DD genotype associated overshoot of blood glucose concentration depended on prior exercise to exhaustion. Interestingly, the blood concentration of glucose is variably affected by different moderate intense training interventions (Álvarez et al., 2017). This seeming disconnection could be related due to the relatively small sample size of the study. Alternatively, it may reflect that the capacity for insulin- and contraction-induced glucose handling is set by the overall surface area of the endothelium as represented by the capillarisation of peripheral tissues, and especially skeletal muscle (Hedman et al., 2001).

Skeletal muscle tissue is the largest contributor to glucose homeostasis, especially during times of elevated physical activity, which increases glucose uptake to skeletal muscle *via* a vasodilatory effect that counteracts angiotensin 2-mediated vasoconstriction being independent of insulin (Korthuis 2011; Richter and Hargreaves, 2013). Interestingly, we identified ACE-I/D genotype associated differences in the phosphorylation of certain regulatory amino acids for key signaling proteins involved in the import of blood-borne glucose into skeletal muscle *via* the post-translational regulation of the facilitative GLUT-4 transporter (i.e., AKT, SRC, HCK, STAT5A and STAT5B, p38α, and EGFR; Figure 4; du Toit and Donner, 2012; Van Epps-Fung et al., 1997, Bengal et al., 2020). Notably, the phosphorylation level for certain of the former signaling molecules (i.e., AKT-pS473, EGFR-pY1086) in exercised muscle post-exercise stood in a considerable linear relationship with the fold changes in blood glucose concentration after one-legged exercise (Table 5, Supplementary Table S1). Our findings are of interest given that they mirror the reported the improvement in glucose handling in skeletal muscle with pharmacological ACE inhibition by NO-dependent effect of bradykinin and/or antagonism of angiotensin action on skeletal muscle which may include elevated GLUT-4 protein expression (Henriksen and Jacob 2003; Stanford and Goodear, 2014). Consequently, the identified ACE-I/D genotype-dependent phosphorylation levels of signaling proteins imply that dysregulation of glucose handling by genetic ACE inhibition (*via* the I-allele) is associated with shifts in the activation of an array of coupled phosphotransfer enzymes.

Especially, the reciprocal ACE I-allele associated levels of S473 and T308 phosphorylation of insulin-receptor-associated protein kinase B (AKT) is of interest (compare Figure 5A vs. Figure 6A), because these amino acids are separately phosphorylated by 3-phosphoinositide-dependent protein kinase 1 and 2 (Shiojima and Walsh, 2002); thus indicating a possibly angiotensin-dependent involvement of both upstream kinases in AKT phosphorylation post exercise. Full activation of AKT requires phosphorylation of T308 and S473 (Alessi et al., 1996). S473 but not T308 phosphorylation of AKT is typically increased upon insulin stimulation and exercise (Treebak et al., 2014; Cartee 2015). Intriguingly, the dephosphorylation of T308 on AKT coincides with the inactivation of the serine/threonine activity of the AKT enzyme (Trotman et al., 2006). In this respect the observed linear relationship between the phosphorylation level of AKT-S473 (r = −0.952, *p* = 0.035), but not T308 (r = 0.348, *p* = 0.618) phosphorylation in m. Vastus lateralis, with the fold changes in RER is of interest as this parameter corresponds to the utilization of substrates during exercise.

Similarly, the correlation between the phosphorylation level of AKT-S473 and capillary-to-fiber ratio in m. Vastus lateralis is intriguing because the latter reflects of the capacity for insulin-mediated glucose uptake in skeletal muscle (Hedman et al., 2001). AKT-S473 phosphorylation is known with angiogenesis (Shiojima and Walsh 2002) and high angiotensin 2 levels are associated with exercise-induced skeletal muscle angiogenesis in laboratory models (reviewed in Rodrigues et al., 2022). As well, we observed a trend for a negative correlation between the phosphorylation of AKT-S473 and COX4I1 transcript levels in m. Vastus lateralis (Table 5) which relate to the recently reported association between AKT-S473 expression and phosphorylation and the protein expression of cytochrome C oxidative subunits in mouse skeletal muscle (Jaiswal et al., 2022). Collectively, our findings point to AKT-S473 phosphorylation as possible gatekeeper of ACE I/D genotype-related differences in the import and combustion of blood-borne substrates in exhaustively exercised skeletal muscle.

The extent to which the former mentioned other signaling factors relate to the detriment of homozygous ACE D-allele carriers in glucose handling during exercise remains to be explored. Possibly this relates to effects on the acute regulation of capillary perfusion *via* a vasoconstrictive mechanism (van Ginkel et al., 2015a) or is reflective of a reduced capacity for perfusion, as indicated by a near trend for an overall reduced muscle capillary-to-fiber ratio (*p* = 0.094). Regarding the former mechanisms, it is of note that ACE activity and the blood concentration of angiotensin 2 are increased in ACE-DD genotypes compared to ACE I-allele carriers (Valdivieso et al., 2017). Correspondingly, we identified ACE-I/D genotype related differences in the phosphorylation of STAT5a-Y694 and STAT5b-Y699 post exercise (Figure 4, Forrester et al., 2018; Haddad et al., 2005; Satou and Gonzalez-Villalobos 2012). Intriguingly, the studied phosphorylation of other proteins being implicated in angiotensin or cytokine signaling in myogenic cells, i.e., STAT2-pY689, STAT6-pY641, FYN-pY420, AMPKα2-pT172, or smooth muscle cells (i.e., PLC-γ1-pY783, Jiang et al., 2017), was not affected in an ACE-I/D genotype dependent manner after one-legged exercise cells (Table 4). Little is known about signaling downstream of angiotensin receptors AT1 or AT2 receptors in skeletal muscle cells, except for the generic phosphorylation of AMPK post-exercise (Surapongchai et al., 2018), and to which degree the observed regulation may reflect constriction of feeder arteries/arterioles that could affect muscle perfusion, or relate to angiogenic and myogenic processes being modified by angiotensin 2 and its related peptide angiotensin 1–7 during exercise (Fernandes et al., 2011; Akhtar et al., 2012; Evangelista 2020).

**TABLE 4:** Exercise-induced alterations of the muscle kinome in dependence of the ACE-I/D genotype.

phospho-protein	time [min]	ACE-DD			ACE-ID/II			ID/DD vs II		ALL		
		Mean	±	SD	Mean	±	SD	p-value (30 or 480)	p-value (30&480)	Mean	±	SD
AKT-pS473	30	6.72	±	0.31	2.41	±	1.01	<0.001	**<0.001**	3.27	±	2.12
	480	6.29	±	0.29	4.14	±	1.99	0.002		4.86	±	1.87
p-value (480 vs 30)		0.581			**<0.001**					0.153		
AKT-pT308	30	0.61	±	0.23	1.29	±	0.57	0.099	**0.048**	1.16	±	0.58
	480	0.35	±	0.13	1.12	±	0.61	0.235		0.66	±	0.67
p-value (480 vs 30)		0.158			0.311					0.418		
AMPKα1-pT183	30	1.24	±	0.08	1.60	±	0.16	0.563	0.503	1.52	±	0.21
	480	1.36	±	0.09	1.62	±	0.75	0.704		1.53	±	0.55
p-value (480 vs 30)		0.876			0.965					0.876		
AMPKα2-pT172	30	3.58	±	0.77	3.78	±	0.48	0.742	0.409	3.74	±	0.43
	480	4.85	±	1.04	3.90	±	1.18	0.157		4.21	±	1.00
p-value (480 vs 30)		0.102			0.805					0.128		0.43
CHK2-pT68	30	2.47	±	0.14	3.02	±	0.16	0.375	0.557	2.91	±	0.28
	480	2.67	±	0.15	2.38	±	0.40	0.667		2.48	±	0.33
p-value (480 vs 30)		0.797			0.183					0.633		
c-JUN-pS63	30	0.80	±	0.32	1.03	±	0.28	0.704	0.557	0.99	±	0.26
	480	0.44	±	0.18	0.75	±	0.48	0.653		0.65	±	0.38
p-value (480 vs 30)		0.647			0.548					0.482		
CREB-pS133	30	1.68	±	0.23	2.01	±	0.14	0.589	0.697	1.94	±	0.19
	480	2.04	±	0.28	2.06	±	0.71	0.973		2.06	±	0.51
p-value (480 vs 30)		0.639			0.907					0.645		
EGFR-pY1086	30	3.56	±	0.42	4.55	±	0.39	0.110	**0.028**	4.35	±	0.55
	480	3.02	±	0.35	4.05	±	0.68	0.128		3.70	±	0.76
p-value (480 vs 30)		0.486			0.293					0.253		
eNOS-pS1177	30	0.41	±	0.16	0.52	±	0.20	0.868	0.786	0.50	±	0.18
	480	0.24	±	0.09	0.38	±	0.21	0.829		0.33	±	0.17
p-value (480 vs 30)		0.820			0.779					0.733		
ERK1/2-pT202/Y204	30	2.35	±	0.26	2.91	±	0.32	0.366	0.364	2.80	±	0.37
&T185/Y187	480	2.01	±	0.22	2.28	±	0.49	0.685		2.19	±	0.38
p-value (480 vs 30)		0.659			0.190					0.288		
FAK-pY397	30	1.10	±	0.04	1.57	±	0.21	0.449	0.503	1.48	±	0.28
	480	1.16	±	0.04	1.31	±	0.25	0.830		1.26	±	0.20
p-value (480 vs 30)		0.939			0.584					0.826		
FGR-pY412	30	0.76	±	0.09	1.61	±	0.52	0.167	0.172	1.44	±	0.59
	480	0.64	±	0.08	1.04	±	0.34	0.556		0.91	±	0.33
p-value (480 vs 30)		0.881			0.231					0.451		
FYN-pY420	30	1.97	±	0.14	3.01	±	0.53	0.094	0.159	2.80	±	0.65
	480	2.19	±	0.16	2.44	±	0.48	0.705		2.36	±	0.37
p-value (480 vs 30)		0.781			0.239					0.704		
GSK3α/β-pS21/S9	30	2.39	±	0.22	3.15	±	0.67	0.216	0.073	3.00	±	0.67
	480	2.10	±	0.19	2.98	±	1.05	0.192		2.69	±	0.90
p-value (480 vs 30)		0.711			0.721					0.616		
HCK- pY411	30	3.56	±	0.62	6.09	±	1.11	<0.001	**0.001**	5.58	±	1.49
	480	4.56	±	0.80	5.11	±	0.99	0.411		4.93	±	0.77
p-value (480 vs 30)		0.199			**0.043**					0.973		
HSP27-pS78/S82	30	3.23	±	1.07	3.00	±	0.41	0.710	0.973	3.05	±	0.37
	480	2.01	±	0.66	2.27	±	0.59	0.701		2.18	±	0.44
p-value (480 vs 30)		0.118			0.124					0.033		
HSP60	30	0.49	±	0.29	0.33	±	0.20	0.797	0.284	0.36	±	0.18
	480	1.19	±	0.70	0.37	±	0.05	0.224		0.64	±	0.48
p-value (480 vs 30)		0.370			0.939					0.421		
JNK-1/2/3	30	1.61	±	0.05	2.14	±	0.40	0.390	0.275	2.04	±	0.42
-pT183/Y185, T221/Y223	480	1.54	±	0.05	2.01	±	0.64	0.488		1.85	±	0.53
p-value (480 vs 30)		0.923			0.775					0.817		
LCK-pY394	30	1.27	±	0.01	1.68	±	0.27	0.501	0.661	1.60	±	0.30
	480	1.25	±	0.01	1.23	±	0.45	0.983		1.24	±	0.32
p-value (480 vs 30)		0.982			0.349					0.611		
LYN-pY397	30	1.99	±	0.35	2.80	±	0.20	0.185	0.423	2.64	±	0.40
	480	2.55	±	0.45	2.47	±	0.31	0.899		2.50	±	0.23
p-value (480 vs 30)		0.467			0.481					0.800		
MSK1/2-pS376/S360	30	3.64	±	0.37	3.79	±	0.24	0.807	0.457	3.76	±	0.21
	480	3.15	±	0.32	3.68	±	1.14	0.433		3.50	±	0.86
p-value (480 vs 30)		0.528			0.814					0.509		
p27-pT198	30	0.25	±	0.14	0.31	±	0.15	0.929	0.852	0.30	±	0.13
	480	0.11	±	0.06	0.22	±	0.20	0.864		0.19	±	0.16
p-value (480 vs 30)		0.854			0.863					0.805		
p38α-pT180/Y182	30	1.41	±	0.09	2.36	±	0.63	0.123	**0.015**	2.17	±	0.69
	480	1.29	±	0.08	2.58	±	1.27	0.057		2.15	±	1.17
p-value (480 vs 30)		0.882			0.644					0.909		
p53-pS15	30	0.36	±	0.06	0.91	±	0.41	0.371	0.310	0.80	±	0.44
	480	0.29	±	0.05	0.66	±	0.58	0.576		0.54	±	0.46
p-value (480 vs 30)		0.926			0.606					0.728		
p53-pS392	30	0.26	±	0.12	0.53	±	0.20	0.666	0.650	0.47	±	0.21
	480	0.13	±	0.06	0.28	±	0.34	0.826		0.23	±	0.25
p-value (480 vs 30)		0.865			0.601					0.676		
p53-pS46	30	0.77	±	0.17	1.24	±	0.37	0.449	0.395	1.14	±	0.38
	480	0.56	±	0.12	0.87	±	0.50	0.644		0.77	±	0.39
p-value (480 vs 30)		0.788			0.446					0.531		
p70S6K-pT389	30	0.36	±	0.27	0.47	±	0.25	0.853	0.708	0.45	±	0.22
	480	0.11	±	0.08	0.33	±	0.33	0.736		0.26	±	0.27
p-value (480 vs 30)		0.747			0.773					0.670		
p70S6K-pT421/S424	30	0.72	±	0.22	1.15	±	0.43	0.483	0.294	1.06	±	0.42
	480	0.46	±	0.14	0.99	±	0.72	0.434		0.81	±	0.59
p-value (480 vs 30)		0.738			0.731					0.643		
PDGF-Rβ-pY751	30	0.82	±	0.18	1.31	±	0.32	0.426	0.542	1.21	±	0.35
	480	1.11	±	0.24	1.17	±	0.65	0.921		1.15	±	0.46
p-value (480 vs 30)		0.707			0.783					0.859		
PLC-γ1-pY783	30	0.46	±	0.12	0.95	±	0.43	0.431	0.370	0.85	±	0.43
	480	0.31	±	0.08	0.65	±	0.53	0.620		0.54	±	0.42
p-value (480 vs 30)		0.849			0.532					0.625		
PRAS40-pT246	30	1.58	±	0.41	2.81	±	0.76	0.046	0.264	2.57	±	0.86
	480	2.30	±	0.60	2.08	±	0.61	0.751		2.15	±	0.45
p-value (480 vs 30)		0.357			0.127					0.989		
PYK2-pY402	30	0.35	±	0.13	0.75	±	0.27	0.515	0.423	0.67	±	0.29
	480	0.20	±	0.08	0.53	±	0.43	0.624		0.42	±	0.36
p-value (480 vs 30)		0.851			0.650					0.691		
RSK1/2/3-pS380/S386/S377	30	0.94	±	0.42	0.92	±	0.31	0.965	0.771	0.92	±	0.27
	480	0.49	±	0.22	0.78	±	0.34	0.664		0.69	±	0.29
p-value (480 vs 30)		0.562			0.781					0.522		
SRC-pY419	30	2.75	±	0.26	3.69	±	0.45	0.127	**0.043**	3.50	±	0.57
	480	2.40	±	0.23	3.32	±	0.36	0.174		3.01	±	0.59
p-value (480 vs 30)		0.656			0.436					0.431		
STAT2-pY689	30	3.08	±	0.73	3.86	±	0.71	0.074	0.796	3.71	±	0.71
	480	4.67	±	1.10	3.72	±	0.98	0.048		4.03	±	0.90
p-value (480 vs 30)		**0.004**			0.663					**0.027**		
STAT3-pS727	30	0.19	±	0.16	0.40	±	0.19	0.735	0.698	0.36	±	0.19
	480	0.05	±	0.04	0.20	±	0.30	0.828		0.15	±	0.23
p-value (480 vs 30)		0.855			0.669					0.705		
STAT3-pY705	30	0.32	±	0.11	0.71	±	0.31	0.522	0.429	0.63	±	0.32
	480	0.19	±	0.07	0.52	±	0.53	0.626		0.41	±	0.42
p-value (480 vs 30)		0.872			0.689					0.729		
STAT5A-pY694	30	1.86	±	0.27	3.26	±	0.79	0.025	**0.022**	2.98	±	0.92
	480	2.30	±	0.34	3.00	±	0.94	0.295		2.77	±	0.78
p-value (480 vs 30)		0.579			0.595					0.845		
STAT5A/B-pY694/Y699	30	0.88	±	0.27	1.77	±	0.42	0.150	0.209	1.59	±	0.54
	480	1.37	±	0.42	1.63	±	0.31	0.700		1.55	±	0.26
p-value (480 vs 30)		0.529			0.770					0.701		
STAT5B-pY699	30	1.33	±	0.29	2.76	±	0.75	0.022	**0.017**	2.47	±	0.91
	480	1.81	±	0.39	2.59	±	1.35	0.249		2.33	±	1.06
p-value (480 vs 30)		0.542			0.723					0.737		
STAT6-pY641	30	1.78	±	0.50	2.94	±	0.63	0.062	0.305	2.71	±	0.75
	480	2.67	±	0.75	2.46	±	1.20	0.749		2.53	±	0.86
p-value (480 vs 30)		0.254			0.314					0.654		
TOR-pS2448	30	2.75	±	0.20	3.13	±	0.05	0.538	0.339	3.05	±	0.17
	480	2.48	±	0.18	2.97	±	0.62	0.463		2.81	±	0.52
p-value (480 vs 30)		0.732			0.750					0.646		
WNK1-pT60	30	0.75	±	0.07	0.56	±	0.16	0.758	0.622	0.60	±	0.16
	480	0.85	±	0.08	0.59	±	0.02	0.699		0.68	±	0.15
p-value (480 vs 30)		0.896			0.948					0.884		
YES-pY426	30	1.94	±	0.19	2.8	±	0.25	0.166	0.343	2.62	±	0.44
	480	2.22	±	0.22	2.23	±	0.49	0.986		2.23	±	0.35
p-value (480 vs 30)		0.713			0.24					0.766		
β-Catenin	30	1.03	±	0.06	1.28	±	0.25	0.687	0.531	1.23	±	0.24
	480	0.95	±	0.06	1.27	±	0.41	0.631		1.16	±	0.34
p-value (480 vs 30)		0.912			0.983					0.916		

*Mean ± SD for the reference related values of assessed 45 phospho-proteins (as related to the signals of the reference spots × 100), and the respective p-values for the post/hoc effects of time and ACE I-allele (i.e., genotype). Bold values indicate effects which were deemed significant at p < 0.05. Phospho-proteins in bold demonstrated genotype effects. ANOVA with post/hoc test of least significant difference.*

**TABLE 5:** Linear relationship between the phosphorylation of signaling proteins and parameters of glucose metabolism.

		*rrm-value*	*p-value*
AKT-pS473	Glucose_fold	0.872	0.024
AKT-pS473	RER_fold_1leg	−0.970	0.001
AKT-pS473	HDL_fold	0.921	0.009
AKT-pS473	capillary_to_fiber_ratio	−0.701	0.080
AKT-pS473	MCSA type I muscle fiber	−0.945	0.004
AKT-pS473	MCSA type II muscle fiber	−0.952	0.003
AKT-pS473	COX4I1	−0.765	0.076
EGFRY-p1086	Glucose_fold	−0.896	0.016
EGFRY-p1086	HDL_fold	−0.831	0.040
EGFRY-p1086	RER_fold1leg	0.740	0.092
EGFRY-p1086	body mass	−0.850	0.016
STAT5A-pY694	body mass	−0.793	0.033

R-value for repeated measures correlations between the phosphorylation level of assessed phospho-proteins ½ and 8 h after one legged-exercise and anatomical sizes, and fold changes in parameters of glucose metabolism. Repeated measures correlations passing a threshold of *p* < 0.10 and |rrm|>0.70.

## Limitations

Some factors may be considered when valuing the interpretation of our data. For instance, the relatively low number of investigated subjects may be considered to represent a limitation for the statistical power not allowing to unfold all the hypothesized differences between the individual ACE-I/D genotypes, such as glycogen depletion (Valdivieso et al., 2017) notably as only three subjects represented homozygous I-allele carriers (i.e., ACE-II). The deployed single cut-off of a 50 ml O_2_ min^−1^ kg^−1^ for VO_2_max, to declare the studied subjects as aerobically fit may also introduce a certain fuzziness in the statistical sizes. However, the thereby declared fit subjects all performed at least 6 h of physical activity per week at an intensity that required an extra cardiovascular effort, and demonstrated elevated muscle levels of mitochondrial marker, the COX4I1 transcript (Desplanches et al., 2014), compared to the unfit subjects. These findings support the reasonability to allocate subjects to groups of aerobically fit and unfit individuals based on the selected VO_2_max-based criteria. As well, the induced systemic metabolic effects during one-legged cycling exercise (i.e., RER and VO_2_ and concentrations of blood metabolites) may be interpreted respective to the typical test situation of a two-legged cycling test to exhaustion that is typically deployed to challenge whole-body metabolism. One may also consider that certain metabolic factors during one-legged exercise may be specifically affected since exercise was carried out in the fasted state when ketone concentration in blood was slightly above the norm value for a fed state (i.e., 0.59 vs. 0.50 mM; Burstal et al., 2018). In this respect, we identified that RER at rest was higher in the fit than unfit subjects before the one-legged exercise when we noted an inverse trend before the two-legged exercise test (compare Tables 1, 2). We choose one-legged exercise as a stimulus to assess the influence of the ACE-I/D genotype, to exclude effects of a limitation of cardiac output, which is associated with the ACE-I/D genotype as well (Hernández et al., 2003; Puthucheary et al., 2011). Nevertheless, the observed depletion of muscle glycogen concentration witnessed the exhaustive nature of the stimulus at the local, muscle level. We also indicate that we did assess the phosphorylation of signaling factors only in samples being collected at two time points post-exercise and did not localize the cell type expressing the studied proteins in which phosphorylation was affected. This was motivated by the fact that the selected sample points are known to reflect active signaling in exercised muscle (Camera et al., 2010; Moberg et al., 2020), and as sufficient sample was only available for these time points.

In order to account for random influences on phosphoprotein levels in the last biopsy 8 h after exercise in the fasted state, care was taken to avoid additional physical activity and provide the subjects with a meal. We can confirm that all subjects exercised in the fasted state based on elevated ketone levels and consumed the provided sandwiches after exercise. However, we cannot exclude a possible interference of dietary factors to the observed variability in phosphoprotein levels 8 h post exercise, because the effective caloric intake after exercise was not standardized. The absence of main effects of time on protein phosphorylation, whereas strong, interacting, effects were identified for the ACE-I/D genotype and factor identity, indicate that influences of one-legged exercise on phosphorylation of the studied signaling factors in vastus lateralis muscle were comparably equal between 0.5 and 8 h post exercise. Interestingly, ACE I-allele associated differences were observed between the two time points, indicating a selective fall in overall phosphorylation levels of studied signaling proteins between 8 and ½ h post exercise in ACE I-allele carriers. The extent to which this relates to the specific stimulus of exhaustive one-legged exercise in the fasted state with a subsequent period of rest under unsolicited food intake remains to be explored. Finally, the identified significant relationships of post exercise phosphorylation levels of ACE I-allele associated glucose signaling proteins with structural and metabolic hallmarks of exercise performance support the relevance of our sample design and point out novel candidates for angiotensin signaling in skeletal muscle.

### Perspectives

Future investigations may explore to which extent angiotensin target pharmacological and physiological interventions are effective in promoting local improvements in aerobic glucose metabolism by enhancing trans-capillary glucose uptake and mitigating inactivity-related type II diabetes (Henriksen and Jacob 2003). These attempts may also explore whether regular exercise or fitness in subject populations, i.e., ACE-DD genotypes, that are at risk of developing this affection (Thomas et al., 2006; Smith et al., 2016; Surapongchai et al., 2018) improves glucose handling.

## Conclusion

Aerobically fit subjects carrying the ACE insertion allele demonstrate an enhanced capacity for post-translational regulation of glucose uptake and angiotensin-mediated vasoconstriction paralleling and correlating with indexes of contraction-induced alterations of glucose handling such as the blood concentration of glucose, respiration exchange ratio, capillary-to-fiber ratio, and muscle glycogen content. An overshoot in the blood concentration of glucose after exhaustive exercise was evident in homozygous ACE D-allele carries but was not affected by the state of aerobic fitness, and physical activity, indicating that an ACE-I/D genotype-related angiogenic mechanism, but not the aerobic fitness state, explain the observed differences in glucose handling in the cardiovasculature and skeletal muscle during exhaustive one-legged exercise.

## Data Availability

The original contributions presented in the study are included in the article/Supplementary Materials, further inquiries can be directed to the corresponding author.
