# Supplementary material for: Post-translational dysregulation of glucose uptake during exhaustive cycling exercise in vastus lateralis muscle of healthy homozygous carriers of the ACE deletion allele
Source: Front Physiol. 2022 Sep 6;13:933792. doi: 10.3389/fphys.2022.933792 (PMC9488703; doi:10.3389/fphys.2022.933792)
Supplement: Supplementary file 1 [file Table1.DOCX]

**Supplemental table 1: *Linear relationship between the assessed parameters.*** R- and p-values for linear relationships from Pearson moment correlations between the phosphorylation level of assessed phosphoproteins ½ and 8 hours after one legged-exercise and the assessed anatomical and physiological parameters before and after one-legged and two-legged cycling exercise. Abbreviations: 1l, one-legged exercise; 2l, two-legged exercise; BMI, body mass index; CD, capillary density; CFR, capillary-to-fiber ratio; COX4I1, cytochrome C oxidase subunit 4 isoform 1 transcript; fold, post vs. pre ratio with one-legged exercise; glu, blood glucose concentration; Gly, muscle glycogen concentration; ht, body height; ket, blood concentration of ketones; LDL, low density lipoprotein concentration in blood; MCSA I, MCSA type I muscle fibers; MCSA II, MCSA type II muscle fibers; P I, percentage type I muscle fibers; Pmax, maximal power during cycling exercise to exhaustion; post, after one-legged exercise; pre, before one-legged exercise; RER, respiration exchange ratio; TAG, triacylglycerol concentration in blood; T Chol, total cholesterol concentration in blood.wt, body weight.

MCSA_I MCSA_II P_I CD CFR COX4I1_pre Gly_fold Glu_fold T_Chol_fold LDL__fold TAG__fold Ketones__fold HDL__fold RER_fold1l RER_fold2l Age Wt Ht BMI Pmax_1l VO2peak_1l Pmax_2l VO2peak_2l ACE_activity ACE_mRNApre AktS473 AktT308 AMPKa2T172 EGFRY1086 FynY420 GSK3abS21_S9 HckY411 HSP27S78_S82 MSK12S376_S360 p38aT180_Y182 PRAS40T246 SrcY419 STAT2Y689 STAT5aY694 STAT5bY699 STAT6Y641

MCSA_I r-value 1.00 0.98 0.66 0.71 0.89 0.72 0.53 -0.95 1.00 1.00 1.00 -1.00 -1.00 0.94 0.00 0.02 -0.25 -0.78 0.92 -0.91 -0.98 -0.49 -0.75 0.48 0.74 -0.97 -0.05 -0.24 0.41 0.08 0.04 0.10 -0.52 -0.21 0.30 -0.14 0.41 -0.07 0.18 0.10 -0.02

p-value NA 0.08 0.44 0.40 0.20 0.10 0.57 0.10 0.00 0.00 0.00 0.00 0.01 0.12 0.00 0.56 0.39 0.31 0.18 0.12 0.03 0.62 0.35 0.62 0.41 0.09 0.50 0.28 0.48 0.39 0.57 0.37 0.58 0.64 0.39 0.20 0.60 0.09 0.38 0.39 0.15

MCSA_II r-value 0.98 1.00 0.49 0.65 0.97 0.73 0.70 -0.89 1.00 1.00 1.00 -1.00 -1.00 0.99 1.00 0.20 -0.37 -0.88 0.96 -0.95 -0.98 -0.53 -0.78 0.47 0.76 -0.99 -0.01 -0.28 0.46 0.08 0.05 0.10 -0.44 -0.03 0.38 -0.20 0.47 -0.09 0.20 0.11 -0.05

p-value 0.08 NA 0.62 0.44 0.09 0.10 0.40 0.23 0.00 0.00 0.00 0.00 0.01 0.03 0.00 0.53 0.42 0.23 0.12 0.12 0.08 0.56 0.32 0.63 0.35 0.03 0.60 0.30 0.55 0.53 0.70 0.51 0.65 0.60 0.47 0.36 0.58 0.13 0.51 0.53 0.28

P_I r-value 0.66 0.49 1.00 0.67 0.27 0.10 -0.18 -0.90 1.00 1.00 1.00 -1.00 -0.68 0.50 0.00 -0.68 0.37 -0.15 0.42 -0.40 -0.57 -0.12 -0.30 0.32 0.60 -0.53 -0.22 -0.15 0.00 -0.04 -0.08 -0.03 -0.57 -0.79 -0.17 -0.01 0.05 -0.16 -0.07 -0.04 -0.09

p-value 0.44 0.62 NA 0.39 0.79 0.86 0.52 0.20 0.00 0.00 0.00 0.00 0.65 1.00 0.00 0.42 0.63 0.87 0.65 0.59 0.53 0.20 0.32 0.41 0.59 0.58 0.34 0.48 0.46 0.18 0.31 0.17 0.50 0.28 0.48 0.16 0.56 0.46 0.28 0.20 0.17

CD r-value 0.71 0.65 0.67 1.00 0.76 0.62 0.41 -0.88 0.05 0.12 0.46 -1.00 -0.64 0.56 0.00 -0.23 -0.32 -0.41 0.29 -0.15 -0.33 0.28 0.05 0.04 0.71 -0.69 -0.03 -0.07 0.62 0.19 0.23 0.31 -0.22 -0.32 -0.03 -0.13 0.51 -0.04 0.47 0.37 0.16

p-value 0.40 0.44 0.39 NA 0.24 0.14 0.39 0.14 0.09 0.24 0.92 0.00 0.65 0.85 0.00 0.61 0.67 0.49 0.28 0.48 0.67 0.54 0.55 0.93 0.37 0.34 0.98 0.26 0.41 0.74 0.77 0.61 0.76 0.64 0.70 0.52 0.55 0.26 0.50 0.58 0.34

CFR r-value 0.89 0.97 0.27 0.76 1.00 0.88 0.85 -0.68 0.12 0.21 0.59 -1.00 -0.67 0.77 1.00 -0.15 -0.40 -0.41 0.20 -0.30 -0.42 -0.17 -0.25 0.41 0.78 -0.80 -0.32 -0.13 0.45 -0.21 -0.16 -0.08 -0.36 -0.09 -0.25 -0.53 0.27 -0.06 0.23 0.00 -0.08

p-value 0.20 0.09 0.79 0.24 NA 0.01 0.16 0.54 0.23 0.43 0.83 0.00 0.57 0.34 0.00 0.85 0.53 0.61 0.19 0.50 0.62 0.84 0.72 0.60 0.27 0.19 0.69 0.19 0.54 0.82 0.83 0.85 0.66 0.82 0.66 0.55 0.80 0.15 0.60 0.78 0.39

COX4I1_pre r-value 0.72 0.73 0.10 0.62 0.88 1.00 0.90 -0.76 -0.23 -0.10 0.53 -0.88 -0.65 0.78 0.77 0.21 -0.29 -0.40 0.19 -0.03 -0.22 -0.06 -0.11 0.40 0.43 -0.74 -0.12 -0.11 0.59 -0.05 0.01 0.18 -0.19 0.13 0.04 -0.32 0.36 0.16 0.60 0.27 0.23

p-value 0.10 0.10 0.86 0.14 0.01 NA 0.01 0.08 0.66 0.86 0.28 0.05 0.17 0.07 0.23 0.65 0.54 0.37 0.69 0.95 0.63 0.89 0.82 0.38 0.34 0.06 0.80 0.81 0.16 0.92 0.98 0.70 0.69 0.78 0.94 0.49 0.43 0.73 0.16 0.56 0.62

Gly_fold r-value 0.53 0.70 -0.18 0.41 0.85 0.90 1.00 -0.43 0.18 0.30 0.68 -1.00 -0.63 0.80 1.00 0.23 -0.44 -0.54 0.35 -0.41 -0.43 -0.36 -0.39 0.36 0.76 -0.65 -0.20 -0.32 0.33 -0.24 -0.15 -0.19 -0.15 0.29 -0.01 -0.61 0.26 -0.23 0.07 -0.13 -0.28

p-value 0.57 0.40 0.52 0.39 0.16 0.01 NA 0.83 0.37 0.59 0.64 0.00 0.68 0.28 0.00 0.75 0.54 0.51 0.34 0.68 0.67 0.61 0.64 0.60 0.33 0.39 0.76 0.53 0.74 0.72 0.66 0.77 0.85 0.59 0.73 0.44 0.68 0.45 0.94 0.83 0.81

Glu_fold r-value -0.95 -0.89 -0.90 -0.88 -0.68 -0.76 -0.43 1.00 -0.65 -0.77 -0.99 1.00 0.91 -0.80 0.00 -0.53 0.82 0.77 -0.62 0.64 0.79 0.41 0.57 -0.51 -0.69 0.91 0.27 0.27 -0.97 0.09 -0.81 0.02 0.66 0.57 -0.66 0.11 -0.87 0.17 -0.91 0.02 0.02

p-value 0.10 0.23 0.20 0.14 0.54 0.08 0.83 NA 0.69 0.45 0.02 0.00 0.10 0.29 0.00 0.92 0.24 0.34 0.70 0.64 0.30 0.77 0.83 0.97 0.62 0.09 0.44 0.44 0.02 0.08 0.26 0.01 0.60 0.82 0.61 0.11 0.15 0.22 0.03 0.01 0.01

T_Chol_fold r-value 1.00 1.00 1.00 0.05 0.12 -0.23 0.18 -0.65 1.00 0.98 0.75 -1.00 -0.68 0.55 0.227 0.86 -0.36 -0.85 0.99 -0.90 -0.84 -0.77 -0.83 0.35 0.34 -0.52 -0.13 -0.83 0.46 -0.08 0.84 -0.15 -0.40 -0.40 0.98 -0.06 0.75 -0.78 -0.19 -0.28 -0.57

p-value 0.00 0.00 0.00 0.09 0.23 0.66 0.37 0.69 NA 0.04 0.50 0.00 0.64 0.90 0.666 0.28 0.72 0.31 0.03 0.20 0.32 0.47 0.34 0.70 0.69 0.97 0.26 0.34 0.92 0.16 0.31 0.31 0.81 0.79 0.05 0.11 0.51 0.44 0.81 0.55 0.86

LDL__fold r-value 1.00 1.00 1.00 0.12 0.21 -0.10 0.30 -0.77 0.98 1.00 0.85 -1.00 -0.80 0.68 0.425 0.94 -0.49 -0.93 1.00 -0.97 -0.93 -0.87 -0.92 0.42 0.42 -0.64 -0.11 -0.77 0.59 -0.04 0.86 -0.07 -0.45 -0.30 1.00 -0.03 0.81 -0.68 0.05 -0.18 -0.44

p-value 0.00 0.00 0.00 0.24 0.43 0.86 0.59 0.45 0.04 NA 0.29 0.00 0.40 0.64 0.401 0.13 0.98 0.14 0.00 0.07 0.15 0.26 0.16 0.84 0.83 0.71 0.23 0.45 0.83 0.07 0.28 0.14 0.90 0.59 0.01 0.06 0.38 0.64 0.95 0.37 0.87

TAG__fold r-value 1.00 1.00 1.00 0.46 0.59 0.53 0.68 -0.99 0.75 0.85 1.00 -1.00 -0.99 0.96 0.92 0.98 -0.85 -0.99 0.85 -0.96 -0.99 -1.00 -0.99 0.63 0.64 -0.93 -0.21 -0.52 0.90 -0.09 0.76 -0.02 -0.59 -0.11 0.86 -0.14 0.87 -0.34 0.67 -0.06 -0.11

p-value 0.00 0.00 0.00 0.92 0.83 0.28 0.64 0.02 0.50 0.29 NA 0.00 0.01 0.09 0.009 0.04 0.31 0.03 0.31 0.08 0.03 0.00 0.02 0.73 0.72 0.14 0.42 0.95 0.21 0.19 0.47 0.03 0.83 0.22 0.27 0.28 0.26 0.68 0.33 0.11 0.21

Ketones__fold r-value -1.00 -1.00 -1.00 -1.00 -1.00 -0.88 -1.00 1.00 -1.00 -1.00 -1.00 1.00 1.00 -1.00 -0.918 -1.00 1.00 1.00 -1.00 1.00 1.00 1.00 1.00 -0.75 -0.76 0.99 0.29 0.31 -0.98 0.07 -0.67 0.00 0.65 0.10 -0.98 0.12 -0.84 0.09 -0.90 0.07 0.00

p-value 0.00 0.00 0.00 0.00 0.00 0.00 0.00 0.00 0.00 0.00 NA 0.00 0.00 0.028 0.00 0.00 0.00 0.00 0.00 0.00 0.00 0.00 0.67 0.65 0.14 0.73 0.75 0.17 0.33 0.78 0.07 0.80 0.41 0.18 0.45 0.53 0.39 0.29 0.33 0.05

HDL__fold r-value -1.00 -1.00 -0.68 -0.64 -0.67 -0.65 -0.63 0.91 -0.68 -0.80 -0.99 1.00 1.00 -0.96 0.00 -0.80 0.90 0.94 -0.76 0.86 0.95 0.71 0.84 -0.62 -0.68 0.96 0.24 0.40 -0.90 0.14 -0.76 0.12 0.63 0.30 -0.79 0.18 -0.87 0.25 -0.68 0.14 0.14

p-value 0.01 0.01 0.65 0.65 0.57 0.17 0.68 0.10 0.64 0.40 0.01 0.00 NA 0.03 0.00 0.28 0.11 0.05 0.37 0.17 0.04 0.48 0.22 0.69 0.64 0.03 0.36 0.76 0.10 0.17 0.38 0.13 0.66 0.50 0.31 0.25 0.16 0.39 0.21 0.17 0.17

RER_fold1l r-value 0.94 0.99 0.50 0.56 0.77 0.78 0.80 -0.80 0.55 0.68 0.96 -1.00 -0.96 1.00 1.00 0.83 -0.96 -0.89 0.67 -0.83 -0.90 -0.80 -0.86 0.72 0.74 -0.95 -0.35 -0.39 0.85 -0.30 0.62 -0.26 -0.64 -0.20 0.69 -0.35 0.77 -0.23 0.55 -0.27 -0.22

p-value 0.12 0.03 1.00 0.85 0.34 0.07 0.28 0.29 0.90 0.64 0.09 0.00 0.03 NA 0.00 0.22 0.03 0.11 0.58 0.23 0.11 0.28 0.17 0.46 0.53 0.04 0.62 0.73 0.18 0.49 0.71 0.42 0.65 0.27 0.52 0.63 0.34 0.35 0.34 0.43 0.33

RER_fold2l r-value 0.00 1.00 0.00 0.00 1.00 0.77 1.00 0.00 1.00 1.00 1.00 -1.00 0.00 1.00 1.00 1.00 -1.00 -1.00 0.00 -1.00 -1.00 -1.00 -1.00 0.75 0.466 -0.45 -0.71 -0.69 0.07 -0.97 0.17 -1.00 -0.47 -0.07 0.25 -0.97 0.25 -0.70 -0.87 -0.96 -1.00

p-value 0.00 0.00 0.00 0.00 0.00 0.23 0.00 0.00 0.227 0.425 0.92 -0.918 0.00 0.00 NA 0.00 0.00 0.00 0.00 0.00 0.00 0.00 0.00 0.67 0.352 0.94 0.73 0.75 0.33 0.23 0.54 0.05 0.96 0.33 0.67 0.23 0.67 0.74 0.33 0.25 0.06

Age r-value 0.02 0.20 -0.68 -0.23 -0.15 0.21 0.23 -0.53 0.666 0.401 0.009 0.028 -0.80 0.83 1.00 1.00 -0.65 -0.62 0.42 -0.18 -0.14 0.01 -0.07 -0.37 -0.06 -0.05 0.72 -0.03 0.49 0.64 0.60 0.55 0.51 0.88 0.89 0.44 0.58 0.11 0.44 0.50 0.32

p-value 0.56 0.53 0.42 0.61 0.85 0.65 0.75 0.92 0.28 0.13 0.04 0.00 0.28 0.22 0.00 NA 0.34 0.41 0.54 0.31 0.35 0.10 0.18 0.44 0.85 0.68 0.27 0.60 0.59 0.37 0.38 0.47 0.54 0.12 0.11 0.64 0.48 0.73 0.43 0.53 0.65

Wt r-value -0.25 -0.37 0.37 -0.32 -0.40 -0.29 -0.44 0.82 -0.36 -0.49 -0.85 1.00 0.90 -0.96 -1.00 -0.65 1.00 0.47 -0.03 -0.12 0.01 -0.32 -0.21 0.23 -0.20 0.36 -0.52 -0.41 -0.89 -0.62 -0.55 -0.71 -0.29 -0.68 -0.50 -0.38 -0.66 -0.57 -0.85 -0.74 -0.71

p-value 0.39 0.42 0.63 0.67 0.53 0.54 0.54 0.24 0.72 0.98 0.31 0.00 0.11 0.03 0.00 0.34 NA 0.55 0.93 0.65 0.53 0.43 0.47 0.32 0.66 0.45 0.54 0.59 0.14 0.40 0.45 0.30 0.37 0.34 0.52 0.69 0.39 0.44 0.12 0.26 0.34

Ht r-value -0.78 -0.88 -0.15 -0.41 -0.41 -0.40 -0.54 0.77 -0.85 -0.93 -0.99 1.00 0.94 -0.89 -1.00 -0.62 0.47 1.00 -0.89 0.74 0.78 0.28 0.53 -0.01 -0.48 0.73 -0.43 0.45 -0.58 -0.46 -0.47 -0.41 -0.03 -0.36 -0.69 -0.08 -0.75 0.29 -0.33 -0.38 -0.01

p-value 0.31 0.23 0.87 0.49 0.61 0.37 0.51 0.34 0.31 0.14 0.03 0.00 0.05 0.11 0.00 0.41 0.55 NA 0.15 0.33 0.31 0.58 0.47 0.87 0.63 0.32 0.55 0.62 0.47 0.52 0.55 0.57 0.90 0.61 0.36 0.48 0.30 0.71 0.69 0.62 0.68

BMI r-value 0.92 0.96 0.42 0.29 0.20 0.19 0.35 -0.62 0.99 1.00 0.85 -1.00 -0.76 0.67 0.00 0.42 -0.03 -0.89 1.00 -0.85 -0.82 -0.40 -0.64 0.04 0.39 -0.58 0.29 -0.72 0.22 0.26 0.31 0.15 -0.03 0.10 0.59 -0.05 0.54 -0.63 -0.03 0.09 -0.33

p-value 0.18 0.12 0.65 0.28 0.19 0.69 0.34 0.70 0.03 0.00 0.31 0.00 0.37 0.58 0.00 0.54 0.93 0.15 NA 0.23 0.24 0.58 0.39 0.84 0.50 0.47 0.54 0.31 0.84 0.58 0.65 0.70 0.85 0.72 0.44 0.37 0.50 0.42 0.95 0.80 0.76

Pmax_1l r-value -0.91 -0.95 -0.40 -0.15 -0.30 -0.03 -0.41 0.64 -0.90 -0.97 -0.96 1.00 0.86 -0.83 -1.00 -0.18 -0.12 0.74 -0.85 1.00 0.97 0.79 0.94 -0.44 -0.49 0.70 0.05 0.69 0.02 0.08 0.07 0.16 0.37 0.06 -0.23 0.25 -0.24 0.57 0.29 0.21 0.46

p-value 0.12 0.12 0.59 0.48 0.50 0.95 0.68 0.64 0.20 0.07 0.08 0.00 0.17 0.23 0.00 0.31 0.65 0.33 0.23 NA 0.02 0.26 0.13 0.65 0.59 0.33 0.37 0.32 0.80 0.42 0.54 0.51 0.71 0.36 0.36 0.23 0.68 0.45 0.77 0.60 0.53

VO2peak_1l r-value -0.98 -0.98 -0.57 -0.33 -0.42 -0.22 -0.43 0.79 -0.84 -0.93 -0.99 1.00 0.95 -0.90 -1.00 -0.14 0.01 0.78 -0.82 0.97 1.00 0.70 0.89 -0.44 -0.55 0.83 0.01 0.57 -0.16 -0.03 -0.02 0.02 0.42 0.10 -0.22 0.17 -0.35 0.43 0.11 0.06 0.28

p-value 0.03 0.08 0.53 0.67 0.62 0.63 0.67 0.30 0.32 0.15 0.03 0.00 0.04 0.11 0.00 0.35 0.53 0.31 0.24 0.02 NA 0.35 0.17 0.65 0.53 0.19 0.36 0.45 0.63 0.35 0.49 0.39 0.67 0.44 0.39 0.16 0.60 0.47 0.58 0.45 0.34

Pmax_2l r-value -0.49 -0.53 -0.12 0.28 -0.17 -0.06 -0.36 0.41 -0.77 -0.87 -1.00 1.00 0.71 -0.80 -1.00 0.01 -0.32 0.28 -0.40 0.79 0.70 1.00 0.95 -0.67 -0.39 0.40 0.39 0.53 0.45 0.51 0.51 0.59 0.47 0.06 0.18 0.48 0.27 0.45 0.63 0.62 0.61

p-value 0.62 0.56 0.20 0.54 0.84 0.89 0.61 0.77 0.47 0.26 0.00 0.00 0.48 0.28 0.00 0.10 0.43 0.58 0.58 0.26 0.35 NA 0.10 0.29 0.70 0.66 0.40 0.52 0.65 0.52 0.56 0.42 0.48 0.05 0.31 0.61 0.72 0.62 0.40 0.39 0.45

VO2peak_2l r-value -0.75 -0.78 -0.30 0.05 -0.25 -0.11 -0.39 0.57 -0.83 -0.92 -0.99 1.00 0.84 -0.86 -1.00 -0.07 -0.21 0.53 -0.64 0.94 0.89 0.95 1.00 -0.61 -0.45 0.60 0.23 0.60 0.23 0.30 0.30 0.37 0.47 0.07 -0.01 0.35 0.01 0.49 0.45 0.41 0.51

p-value 0.35 0.32 0.32 0.55 0.72 0.82 0.64 0.83 0.34 0.16 0.02 0.00 0.22 0.17 0.00 0.18 0.47 0.47 0.39 0.13 0.17 0.10 NA 0.43 0.65 0.45 0.33 0.46 0.71 0.42 0.54 0.52 0.59 0.17 0.29 0.44 0.63 0.60 0.64 0.59 0.53

ACE_activity r-value 0.48 0.47 0.32 0.04 0.41 0.40 0.36 -0.51 0.35 0.42 0.63 -0.75 -0.62 0.72 0.75 -0.37 0.23 -0.01 0.04 -0.44 -0.44 -0.67 -0.61 1.00 0.75 -0.50 -0.82 0.01 -0.23 -0.68 -0.82 -0.57 -0.93 -0.13 -0.56 -0.63 -0.56 -0.03 -0.54 -0.64 -0.38

p-value 0.62 0.63 0.41 0.93 0.60 0.38 0.60 0.97 0.70 0.84 0.73 0.67 0.69 0.46 0.67 0.44 0.32 0.87 0.84 0.65 0.65 0.29 0.43 NA 0.34 0.53 0.15 0.41 0.49 0.31 0.19 0.44 0.04 0.09 0.50 0.42 0.49 0.42 0.51 0.37 0.56

ACE_mRNApre r-value 0.74 0.76 0.60 0.71 0.78 0.43 0.76 -0.69 0.34 0.42 0.64 -0.76 -0.68 0.74 0.466 -0.06 -0.20 -0.48 0.39 -0.49 -0.55 -0.39 -0.45 0.75 1.00 -0.83 -0.52 -0.04 0.43 -0.33 -0.44 -0.16 -0.72 0.18 -0.17 -0.64 -0.03 -0.05 -0.05 -0.24 -0.11

p-value 0.41 0.35 0.59 0.37 0.27 0.34 0.33 0.62 0.69 0.83 0.72 0.65 0.64 0.53 0.352 0.85 0.66 0.63 0.50 0.59 0.53 0.70 0.65 0.34 NA 0.18 0.56 0.09 0.60 0.74 0.60 0.83 0.36 0.45 0.85 0.46 0.70 0.12 0.83 0.82 0.39

AktS473 r-value -0.97 -0.99 -0.53 -0.69 -0.80 -0.74 -0.65 0.91 -0.52 -0.64 -0.93 0.99 0.96 -0.95 -0.45 -0.05 0.36 0.73 -0.58 0.70 0.83 0.40 0.60 -0.50 -0.83 1.00 0.13 0.23 -0.52 -0.05 0.00 -0.13 0.52 0.04 -0.06 0.24 -0.42 0.10 -0.22 -0.13 0.01

p-value 0.09 0.03 0.58 0.34 0.19 0.06 0.39 0.09 0.97 0.71 0.14 0.14 0.03 0.04 0.94 0.68 0.45 0.32 0.47 0.33 0.19 0.66 0.45 0.53 0.18 NA 0.73 0.21 0.48 0.54 0.76 0.46 0.51 0.60 0.80 0.37 0.58 0.11 0.48 0.49 0.22

AktT308 r-value -0.05 -0.01 -0.22 -0.03 -0.32 -0.12 -0.20 0.27 -0.13 -0.11 -0.21 0.29 0.24 -0.35 -0.71 0.72 -0.52 -0.43 0.29 0.05 0.01 0.39 0.23 -0.82 -0.52 0.13 1.00 -0.02 0.50 0.92 0.96 0.81 0.78 0.41 0.87 0.81 0.81 0.14 0.69 0.82 0.55

p-value 0.50 0.60 0.34 0.98 0.69 0.80 0.76 0.44 0.26 0.23 0.42 0.73 0.36 0.62 0.73 0.27 0.54 0.55 0.54 0.37 0.36 0.40 0.33 0.15 0.56 0.73 NA 0.47 0.58 0.05 0.07 0.14 0.18 0.40 0.16 0.25 0.27 0.60 0.31 0.14 0.52

AMPKa2T172 r-value -0.24 -0.28 -0.15 -0.07 -0.13 -0.11 -0.32 0.27 -0.83 -0.77 -0.52 0.31 0.40 -0.39 -0.69 -0.03 -0.41 0.45 -0.72 0.69 0.57 0.53 0.60 0.01 -0.04 0.23 -0.02 1.00 0.35 0.22 -0.01 0.39 -0.12 0.28 -0.15 0.41 -0.21 0.96 0.43 0.36 0.77

p-value 0.28 0.30 0.48 0.26 0.19 0.81 0.53 0.44 0.34 0.45 0.95 0.75 0.76 0.73 0.75 0.60 0.59 0.62 0.31 0.32 0.45 0.52 0.46 0.41 0.09 0.21 0.47 NA 0.60 0.77 0.65 0.67 0.34 0.81 0.59 0.52 0.80 0.03 0.59 0.69 0.27

EGFRY1086 r-value 0.41 0.46 0.00 0.62 0.45 0.59 0.33 -0.97 0.46 0.59 0.90 -0.98 -0.90 0.85 0.07 0.49 -0.89 -0.58 0.22 0.02 -0.16 0.45 0.23 -0.23 0.43 -0.52 0.50 0.35 1.00 0.72 0.63 0.83 0.13 0.45 0.54 0.42 0.76 0.47 0.90 0.83 0.71

p-value 0.48 0.55 0.46 0.41 0.54 0.16 0.74 0.02 0.92 0.83 0.21 0.17 0.10 0.18 0.33 0.59 0.14 0.47 0.84 0.80 0.63 0.65 0.71 0.49 0.60 0.48 0.58 0.60 NA 0.32 0.41 0.22 0.36 0.64 0.51 0.58 0.25 0.50 0.16 0.22 0.35

FynY420 r-value 0.08 0.08 -0.04 0.19 -0.21 -0.05 -0.24 0.09 -0.08 -0.04 -0.09 0.07 0.14 -0.30 -0.97 0.64 -0.62 -0.46 0.26 0.08 -0.03 0.51 0.30 -0.68 -0.33 -0.05 0.92 0.22 0.72 1.00 0.95 0.97 0.54 0.40 0.83 0.88 0.81 0.35 0.83 0.95 0.75

p-value 0.39 0.53 0.18 0.74 0.82 0.92 0.72 0.08 0.16 0.07 0.19 0.33 0.17 0.49 0.23 0.37 0.40 0.52 0.58 0.42 0.35 0.52 0.42 0.31 0.74 0.54 0.05 0.77 0.32 NA 0.08 0.01 0.47 0.39 0.18 0.22 0.26 0.72 0.15 0.03 0.29

GSK3abS21_S9 r-value 0.04 0.05 -0.08 0.23 -0.16 0.01 -0.15 -0.81 0.84 0.86 0.76 -0.67 -0.76 0.62 0.17 0.60 -0.55 -0.47 0.31 0.07 -0.02 0.51 0.30 -0.82 -0.44 0.00 0.96 -0.01 0.63 0.95 1.00 0.87 0.72 0.29 0.82 0.78 0.89 0.13 0.79 0.90 0.59

p-value 0.57 0.70 0.31 0.77 0.83 0.98 0.66 0.26 0.31 0.28 0.47 0.78 0.38 0.71 0.54 0.38 0.45 0.55 0.65 0.54 0.49 0.56 0.54 0.19 0.60 0.76 0.07 0.65 0.41 0.08 NA 0.13 0.29 0.33 0.16 0.30 0.19 0.78 0.17 0.10 0.46

HckY411 r-value 0.10 0.10 -0.03 0.31 -0.08 0.18 -0.19 0.02 -0.15 -0.07 -0.02 0.00 0.12 -0.26 -1.00 0.55 -0.71 -0.41 0.15 0.16 0.02 0.59 0.37 -0.57 -0.16 -0.13 0.81 0.39 0.83 0.97 0.87 1.00 0.41 0.39 0.73 0.84 0.77 0.51 0.90 0.98 0.85

p-value 0.37 0.51 0.17 0.61 0.85 0.70 0.77 0.01 0.31 0.14 0.03 0.07 0.13 0.42 0.05 0.47 0.30 0.57 0.70 0.51 0.39 0.42 0.52 0.44 0.83 0.46 0.14 0.67 0.22 0.01 0.13 NA 0.41 0.38 0.29 0.24 0.29 0.53 0.09 0.02 0.21

HSP27S78_S82 r-value -0.52 -0.44 -0.57 -0.22 -0.36 -0.19 -0.15 0.66 -0.40 -0.45 -0.59 0.65 0.63 -0.64 -0.47 0.51 -0.29 -0.03 -0.03 0.37 0.42 0.47 0.47 -0.93 -0.72 0.52 0.78 -0.12 0.13 0.54 0.72 0.41 1.00 0.30 0.56 0.47 0.51 -0.05 0.43 0.48 0.24

p-value 0.58 0.65 0.50 0.76 0.66 0.69 0.85 0.60 0.81 0.90 0.83 0.80 0.66 0.65 0.96 0.54 0.37 0.90 0.85 0.71 0.67 0.48 0.59 0.04 0.36 0.51 0.18 0.34 0.36 0.47 0.29 0.41 NA 0.26 0.50 0.62 0.55 0.36 0.49 0.52 0.40

MSK12S376_S360 r-value -0.21 -0.03 -0.79 -0.32 -0.09 0.13 0.29 0.57 -0.40 -0.30 -0.11 0.10 0.30 -0.20 -0.07 0.88 -0.68 -0.36 0.10 0.06 0.10 0.06 0.07 -0.13 0.18 0.04 0.41 0.28 0.45 0.40 0.29 0.39 0.30 1.00 0.62 0.25 0.26 0.35 0.34 0.33 0.35

p-value 0.64 0.60 0.28 0.64 0.82 0.78 0.59 0.82 0.79 0.59 0.22 0.41 0.50 0.27 0.33 0.12 0.34 0.61 0.72 0.36 0.44 0.05 0.17 0.09 0.45 0.60 0.40 0.81 0.64 0.39 0.33 0.38 0.26 NA 0.42 0.53 0.63 0.73 0.37 0.31 0.55

p38aT180_Y182 r-value 0.30 0.38 -0.17 -0.03 -0.25 0.04 -0.01 -0.66 0.98 1.00 0.86 -0.98 -0.79 0.69 0.25 0.89 -0.50 -0.69 0.59 -0.23 -0.22 0.18 -0.01 -0.56 -0.17 -0.06 0.87 -0.15 0.54 0.83 0.82 0.73 0.56 0.62 1.00 0.62 0.75 -0.02 0.51 0.67 0.35

p-value 0.39 0.47 0.48 0.70 0.66 0.94 0.73 0.61 0.05 0.01 0.27 0.18 0.31 0.52 0.67 0.11 0.52 0.36 0.44 0.36 0.39 0.31 0.29 0.50 0.85 0.80 0.16 0.59 0.51 0.18 0.16 0.29 0.50 0.42 NA 0.42 0.29 0.69 0.53 0.36 0.74

PRAS40T246 r-value -0.14 -0.20 -0.01 -0.13 -0.53 -0.32 -0.61 0.11 -0.06 -0.03 -0.14 0.12 0.18 -0.35 -0.97 0.44 -0.38 -0.08 -0.05 0.25 0.17 0.48 0.35 -0.63 -0.64 0.24 0.81 0.41 0.42 0.88 0.78 0.84 0.47 0.25 0.62 1.00 0.49 0.52 0.65 0.81 0.80

p-value 0.20 0.36 0.16 0.52 0.55 0.49 0.44 0.11 0.11 0.06 0.28 0.45 0.25 0.63 0.23 0.64 0.69 0.48 0.37 0.23 0.16 0.61 0.44 0.42 0.46 0.37 0.25 0.52 0.58 0.22 0.30 0.24 0.62 0.53 0.42 NA 0.49 0.54 0.39 0.26 0.18

SrcY419 r-value 0.41 0.47 0.05 0.51 0.27 0.36 0.26 -0.87 0.75 0.81 0.87 -0.84 -0.87 0.77 0.25 0.58 -0.66 -0.75 0.54 -0.24 -0.35 0.27 0.01 -0.56 -0.03 -0.42 0.81 -0.21 0.76 0.81 0.89 0.77 0.51 0.26 0.75 0.49 1.00 -0.02 0.78 0.81 0.43

p-value 0.60 0.58 0.56 0.55 0.80 0.43 0.68 0.15 0.51 0.38 0.26 0.53 0.16 0.34 0.67 0.48 0.39 0.30 0.50 0.68 0.60 0.72 0.63 0.49 0.70 0.58 0.27 0.80 0.25 0.26 0.19 0.29 0.55 0.63 0.29 0.49 NA 0.89 0.27 0.25 0.56

STAT2Y689 r-value -0.07 -0.09 -0.16 -0.04 -0.06 0.16 -0.23 0.17 -0.78 -0.68 -0.34 0.09 0.25 -0.23 -0.70 0.11 -0.57 0.29 -0.63 0.57 0.43 0.45 0.49 -0.03 -0.05 0.10 0.14 0.96 0.47 0.35 0.13 0.51 -0.05 0.35 -0.02 0.52 -0.02 1.00 0.57 0.50 0.87

p-value 0.09 0.13 0.46 0.26 0.15 0.73 0.45 0.22 0.44 0.64 0.68 0.39 0.39 0.35 0.74 0.73 0.44 0.71 0.42 0.45 0.47 0.62 0.60 0.42 0.12 0.11 0.60 0.03 0.50 0.72 0.78 0.53 0.36 0.73 0.69 0.54 0.89 NA 0.45 0.54 0.17

STAT5aY694 r-value 0.18 0.20 -0.07 0.47 0.23 0.60 0.07 -0.91 -0.19 0.05 0.67 -0.90 -0.68 0.55 -0.87 0.44 -0.85 -0.33 -0.03 0.29 0.11 0.63 0.45 -0.54 -0.05 -0.22 0.69 0.43 0.90 0.83 0.79 0.90 0.43 0.34 0.51 0.65 0.78 0.57 1.00 0.96 0.86

p-value 0.38 0.51 0.28 0.50 0.60 0.16 0.94 0.03 0.81 0.95 0.33 0.29 0.21 0.34 0.33 0.43 0.12 0.69 0.95 0.77 0.58 0.40 0.64 0.51 0.83 0.48 0.31 0.59 0.16 0.15 0.17 0.09 0.49 0.37 0.53 0.39 0.27 0.45 NA 0.05 0.23

STAT5bY699 r-value 0.10 0.11 -0.04 0.37 0.00 0.27 -0.13 0.02 -0.28 -0.18 -0.06 0.07 0.14 -0.27 -0.96 0.50 -0.74 -0.38 0.09 0.21 0.06 0.62 0.41 -0.64 -0.24 -0.13 0.82 0.36 0.83 0.95 0.90 0.98 0.48 0.33 0.67 0.81 0.81 0.50 0.96 1.00 0.86

p-value 0.39 0.53 0.20 0.58 0.78 0.56 0.83 0.01 0.55 0.37 0.11 0.33 0.17 0.43 0.25 0.53 0.26 0.62 0.80 0.60 0.45 0.39 0.59 0.37 0.82 0.49 0.14 0.69 0.22 0.03 0.10 0.02 0.52 0.31 0.36 0.26 0.25 0.54 0.05 NA 0.21

STAT6Y641 r-value -0.02 -0.05 -0.09 0.16 -0.08 0.23 -0.28 0.02 -0.57 -0.44 -0.11 0.00 0.14 -0.22 -1.00 0.32 -0.71 -0.01 -0.33 0.46 0.28 0.61 0.51 -0.38 -0.11 0.01 0.55 0.77 0.71 0.75 0.59 0.85 0.24 0.35 0.35 0.80 0.43 0.87 0.86 0.86 1.00

p-value 0.15 0.28 0.17 0.34 0.39 0.62 0.81 0.01 0.86 0.87 0.21 0.05 0.17 0.33 0.06 0.65 0.34 0.68 0.76 0.53 0.34 0.45 0.53 0.56 0.39 0.22 0.52 0.27 0.35 0.29 0.46 0.21 0.40 0.55 0.74 0.18 0.56 0.17 0.23 0.21 NA
