# Supplementary figures and images for: Post-translational dysregulation of glucose uptake during exhaustive cycling exercise in vastus lateralis muscle of healthy homozygous carriers of the ACE deletion allele
Source: Front Physiol. 2022 Sep 6;13:933792. doi: 10.3389/fphys.2022.933792 (PMC9488703; doi:10.3389/fphys.2022.933792)

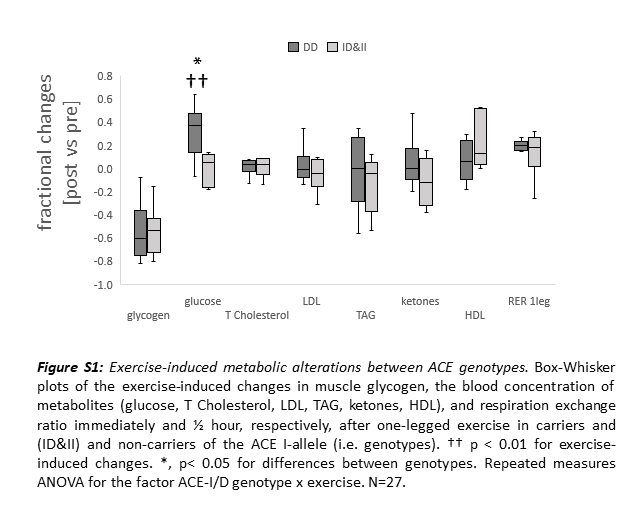

Supplement: Supplementary file 2 [file Image1.tif]
